# A magnetic copper organic framework material as an efficient and recyclable catalyst for the synthesis of 1,2,3-triazole derivatives

**DOI:** 10.1038/s41598-021-00012-3

**Published:** 2021-10-15

**Authors:** Elham Arefi, Amir Khojastehnezhad, Ali Shiri

**Affiliations:** Department of Chemistry, Faculty of Science, Ferdowsi University of Mashhad, Mashhad, Iran

**Keywords:** Chemistry, Catalysis, Green chemistry, Materials chemistry, Organic chemistry

## Abstract

In this study, a core–shell magnetic metal organic framework (MOF) catalyst was introduced based on Fe_3_O_4_ magnetic nanoparticles (MNPs) and copper organic frameworks. In this catalyst, Fe_3_O_4_ MNPs have been coated with MOFs in which copper was the inorganic nodes and 1,3,5-benzenetricarboxylic acid was the organic linkers. Then, the core–shell structures and catalytic efficiency have been confirmed properly and completely with various analyses such as FT-IR, TEM, SEM, TEM mapping, SEM mapping, EDX, PXRD, TGA, ICP and VSM. The Cu moieties in MOF and shell structures can catalyze the synthesis of 1,2,3-triazole derivatives with good to excellent yields in the presence of water as a green solvent. Moreover, this catalyst showed the high reusability due to the super paramagnetic properties.

## Introduction

One of the best nanomaterials that have been recently gained huge attention for immobilization of different organic and inorganic ligands are MNPs and especially Fe_3_O_4_^1–7^. Due to the nanostructures, particle size, and morphology, they showed high magnetic properties, however, these magnetic capacities cause the serious aggregation that is one of the most significant restrictions^8,9^. For problem-solving, design the magnetic supports with core–shell structures can prepare the high-dispersed MNPs^10–16^. Thus, the best strategy is the coating MNPs with various inorganic and organic substances including carbon based materials, silica, alumina, polymers, and MOFs^17–21^. These nanostructures can be considered as shell structures to decrease the aggregation of MNPs.

MOFs are a unique types of crystalline substances with network and porous structures^22^. They have huge internal surface areas (beyond 6000 m^2^/g) and large density of active sites^23,24^. During the last decade, extensive research attempts have been devoted for the preparation of various MOFs and investigations of their application in different fields of science such as, energy storages, biomedical technology, environmental pollution, sensing platforms and catalysis^25–31^. Recently, due to the aforementioned properties, these great porous materials have been attracted chemist’s attention in the term of heterogeneous catalysis^32–34^.

Copper azide–alkyne (CuAAC) cycloaddition reaction is a famous 1,3-dipolar cycloaddition in which the 1,2,3-triazole derivatives are produced with high selectivity^35–38^. The synthesis of these 5-membered compounds is very interesting in organic synthesis and this reaction is the core of many synthetic routes for total synthesis^39,40^. During the past decades, this reaction has been investigated with different kinds of Cu catalysts such as CuI^41^, Cu(OAc)_2_^42^, CuO^43^, CuCl_2_.2H_2_O^44^ and CuSO_4_.5H_2_O^45^, Fe_3_O_4_ NPs supported Cu (II)-β-cyclodextrin^46^, Fe_3_O_4_@SiO_2_ picolinimidoamide-Cu(II) complex^47^, Cu(II) porphyrin graphene oxide^48^, and Cu(II)-supported graphene quantum dots^49^. However, most of the abovementioned Cu catalysts suffered from some sever problems like expensive catalyst preparation methods, using the toxic solvents and reagents, and more importantly difficult catalyst separation approaches. Therefore, present the new and applicable Cu catalyst is a pressing need in organic synthesis and highly recommended for the synthesis of 1,2,3-triazole derivatives.

**Figure 1:**
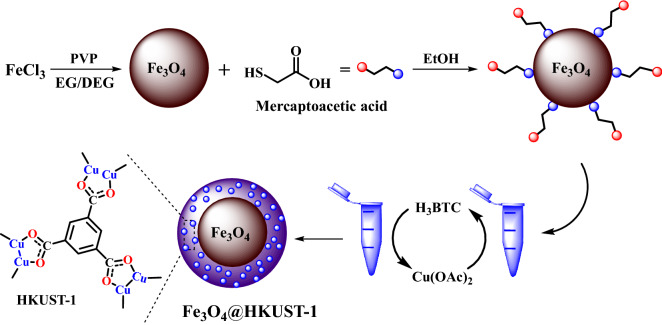
Synthesis of catalyst (Fe_3_O_4_@HKUST-1).

**Figure 2:**
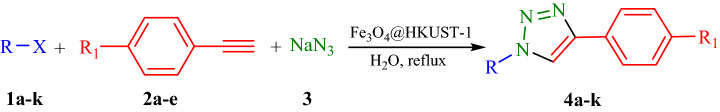
Synthesis of 1,2,3-triazole derivatives in presence of catalyst (Fe_3_O_4_@HKUST-1).

Hence, in this research and in continuation of research programs to develop the organic synthesis and catalysis^50–53^, a new magnetic MOF catalyst has been prepared with core–shell structure. So, initially the Fe_3_O_4_ MNPs have been prepared in the nano dimensions and then, they have been coated with MOFs with Cu as inorganic nodes and 1,3,5-benzenetricarboxylic acid as organic linkers ([Cu_3_(btc)_2_] btc = benzene-1,3,5-tricarboxylic acid, HKUST-1)^54^ (Fig. 1). This easy prepared nanocatalyst can catalyze the synthesis of 1,2,3-triazole derivatives with good to excellent yields and mild reaction conditions (Fig. 2). It is prime importance to note that, to the best of our knowledge, there are no publications for the synthesis 1,2,3-triazole derivatives with the use of magnetic MOFs based on Cu catalyst.

## Results and discussion

In this research, a magnetic metal organic framework with core–shell structure based on HKUST-1 MOF has been developed as an efficient and recyclable catalyst (Fig. 1). Hence, firstly, the Fe_3_O_4_ MNPs have been prepared by using FeCl_3_ and hydrothermal process^55^, and then, the surface of these MNPs has been modified by mercaptoacetic acid and finally, these modified MNPs have been coated with HKUST-1 MOF by a versatile layer-by-layer assembly method and using copper acetate and 1,3,5-benzenetricarboxylic acid (H_3_-BTC) (Fig. 1)^56^. The catalyst structure was characterized with different techniques including, TEM, SEM, TEM mapping, SEM mapping, EDX, TGA, PXRD, VSM, and ICP.

**Figure 3:**
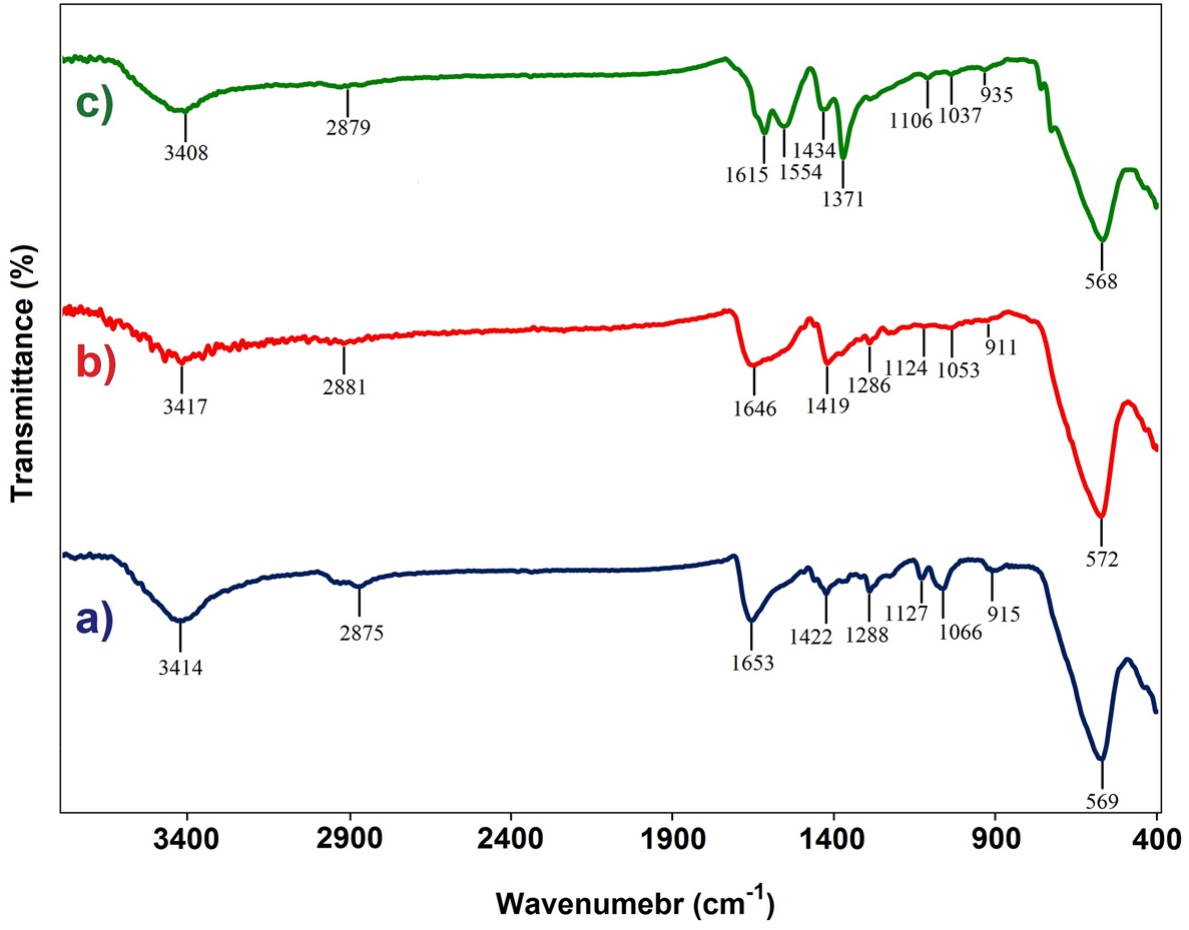
FT‐IR spectra of (**a**) Fe_3_O_4_ (**b**) Fe_3_O_4_-mercaptoacetic acid and (**c**) of Fe_3_O_4_@HKUST-1.

At first, all steps for the preparation of magnetic core–shell (Fe_3_O_4_@HKUST-1) catalyst have been studied by the FT-IR spectroscopy (Fig. 3). In the first spectrum related to Fe_3_O_4_ MNPs (Fig. 3a), there are two distinct sharp peaks at 3414 and 569 cm^−1^ that are attributed to the O–H stretching bands of the hydroxyl functional groups on the surface of MNPs and Fe–O bonds into the structure of MNPs, respectively^55^. In the next spectrum correspond to modified MNPs with mercaptoacetic acid (Fig. 3b), there are not any significant differences between this spectrum with previous one and approximately all peaks are retained with moderate shifts. But in the last spectrum of Fe_3_O_4_@HKUST-1 (Fig. 3c), there are some new peaks including two characteristic peaks at 1615 and 1554 cm^−1^ that can be assigned to the asymmetric stretching vibrations of − COO- and two sharp peaks at 1434 and 1371 cm^−1^ can be ascribed to the symmetric vibrations of − COO^−^^56,57^.

**Figure 4:**
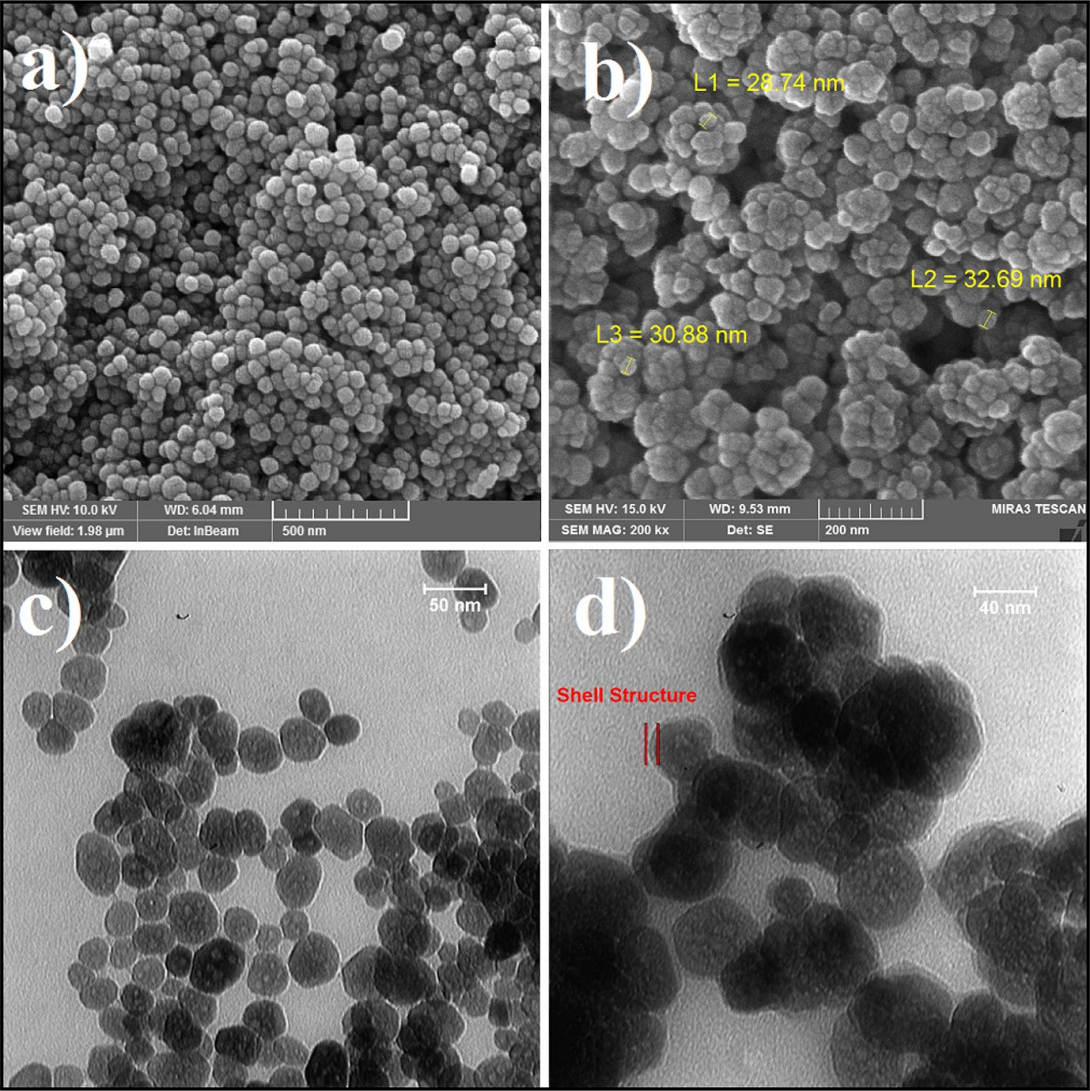
SEM (**a**) and TEM (**c**) images of Fe_3_O_4_ and SEM (**b**) and TEM (**d**) images of Fe_3_O_4_@HKUST-1.

**Figure 5:**
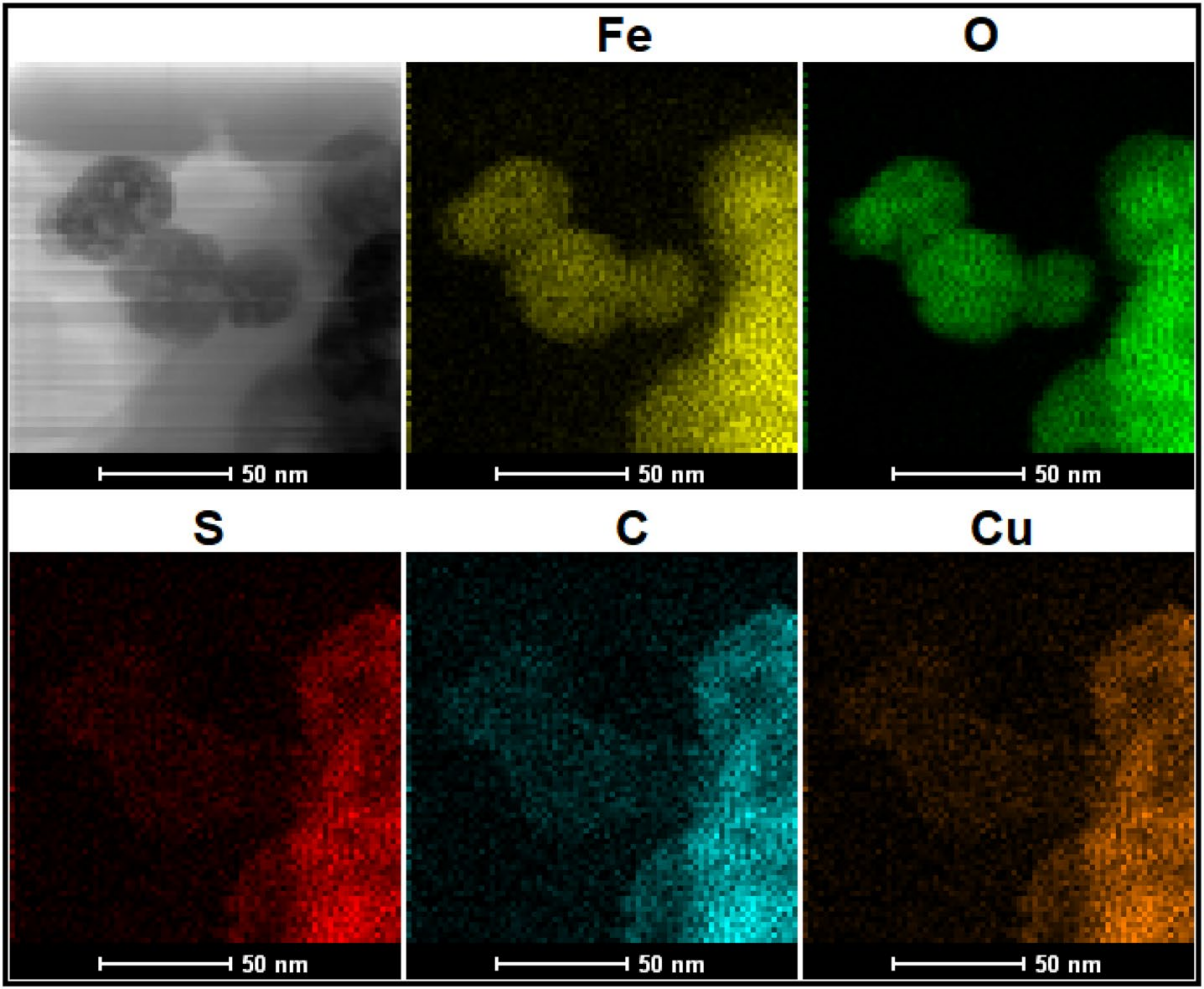
TEM mapping of Fe_3_O_4_@HKUST-1.

**Figure 6:**
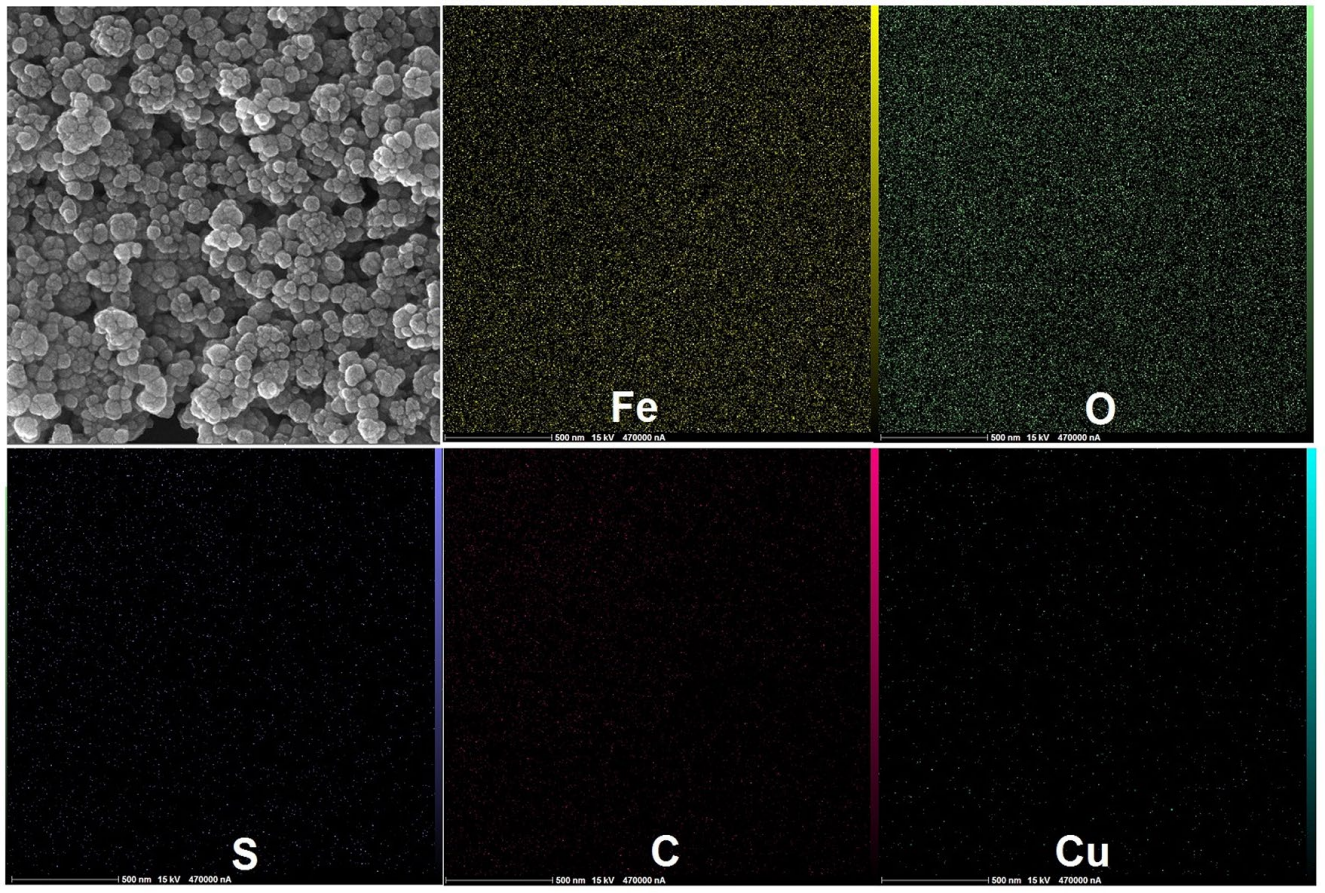
SEM mapping of Fe_3_O_4_@HKUST-1.

SEM and TEM techniques are the important analysis for evaluation of core–shell structures and investigation of morphology and size of NPs. Thereby, SEM and TEM images of bare Fe_3_O_4_ and core–shell Fe_3_O_4_@HKUST-1 MNPs (Final catalyst) have been provided (Fig. 4). As it is evident from SEM images (Fig. 4a and b), the morphology of MNPs is spherical and it has been somewhat changed after coating process (Fig. 4b). Also, the size of bare Fe_3_O_4_ and core–shell Fe_3_O_4_@HKUST-1 MNPs is below 50 nm. Besides, the core–shell structures are confirmed properly with TEM images (Fig. 4d)^19^. Also, the excellent analysis to approve the core–shell structures and elemental analysis is TEM mapping (Fig. 5). This figure obviously showed the core–shell structures of magnetic MOF with all elements including the iron and oxygen for core moieties and sulfur, carbon, and cupper related to the shell parts. As we can see in this image, the morphology of core parts (Fe, O) is changed after coating with MOF (S, C, Cu). Another great analysis to confirm the structure is SEM mapping. As shown in Fig. 6, all elements of catalyst including Fe, O, S, C and Cu are existed in this figure. This figure showed that in addition to iron and oxygen that are the elements of Fe_3_O_4_ MNPs, sulfur and particularly carbon, and copper are existed that are the main elements of shell structures. These outcomes approve the successful formation of core–shell structures.

**Figure 7:**
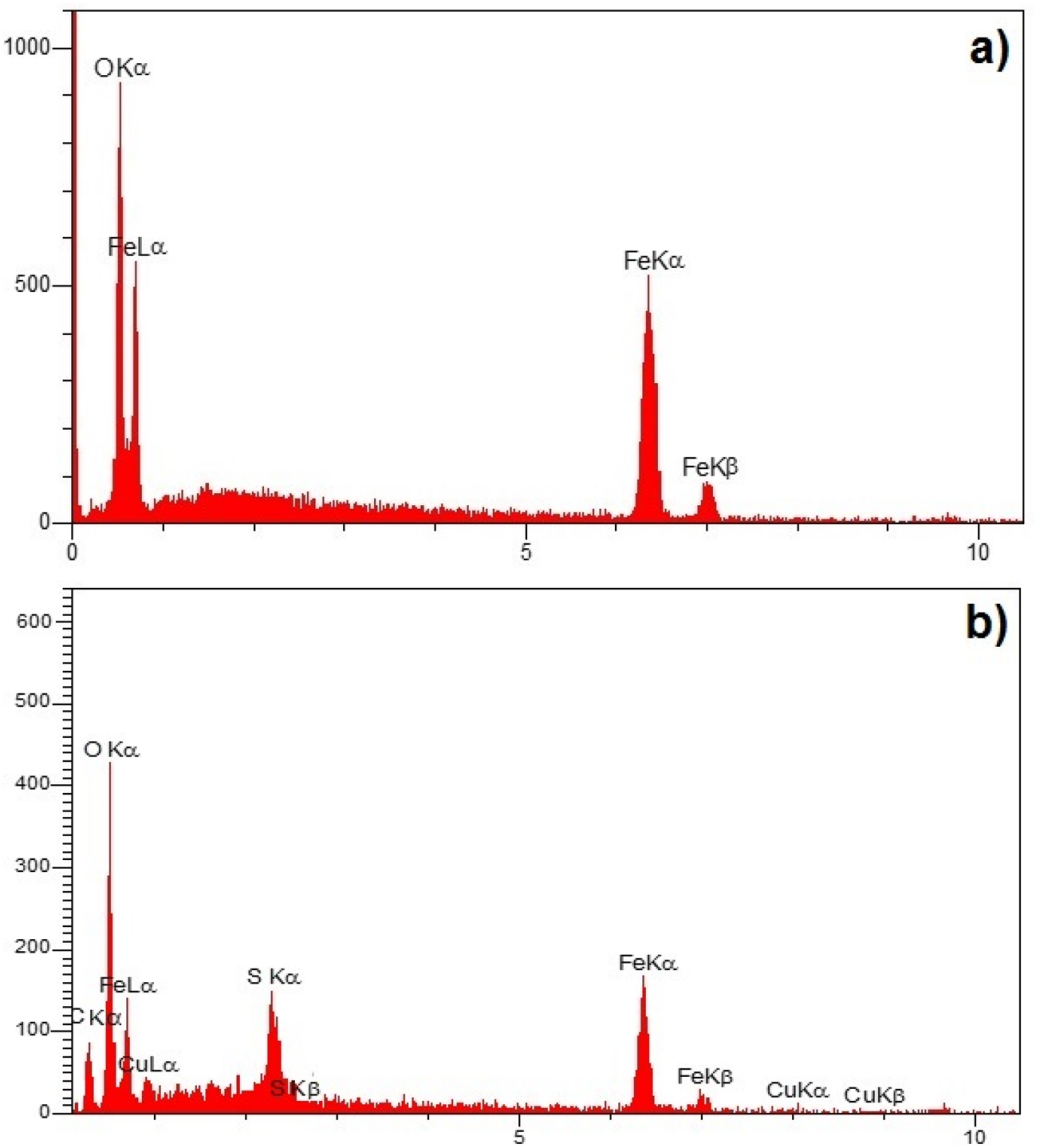
EDX patterns of (**a**) Fe_3_O_4_ and (**b**) Fe_3_O_4_@HKUST-1.

EDX analysis was also performed to study again the elemental analysis. So, this analysis was investigated for both bare Fe_3_O_4_ and core–shell Fe_3_O_4_@HKUST-1 MNPs (Fig. 7). As can be seen in the first pattern (Fig. 7a) correspond to Fe_3_O_4_ MNPs, the iron and oxygen are presented, while in the second pattern (Fig. 7b), in addition to Fe and O, some new elements related to shell structures including sulfur, carbon and more importantly copper are observed.

**Figure 8:**
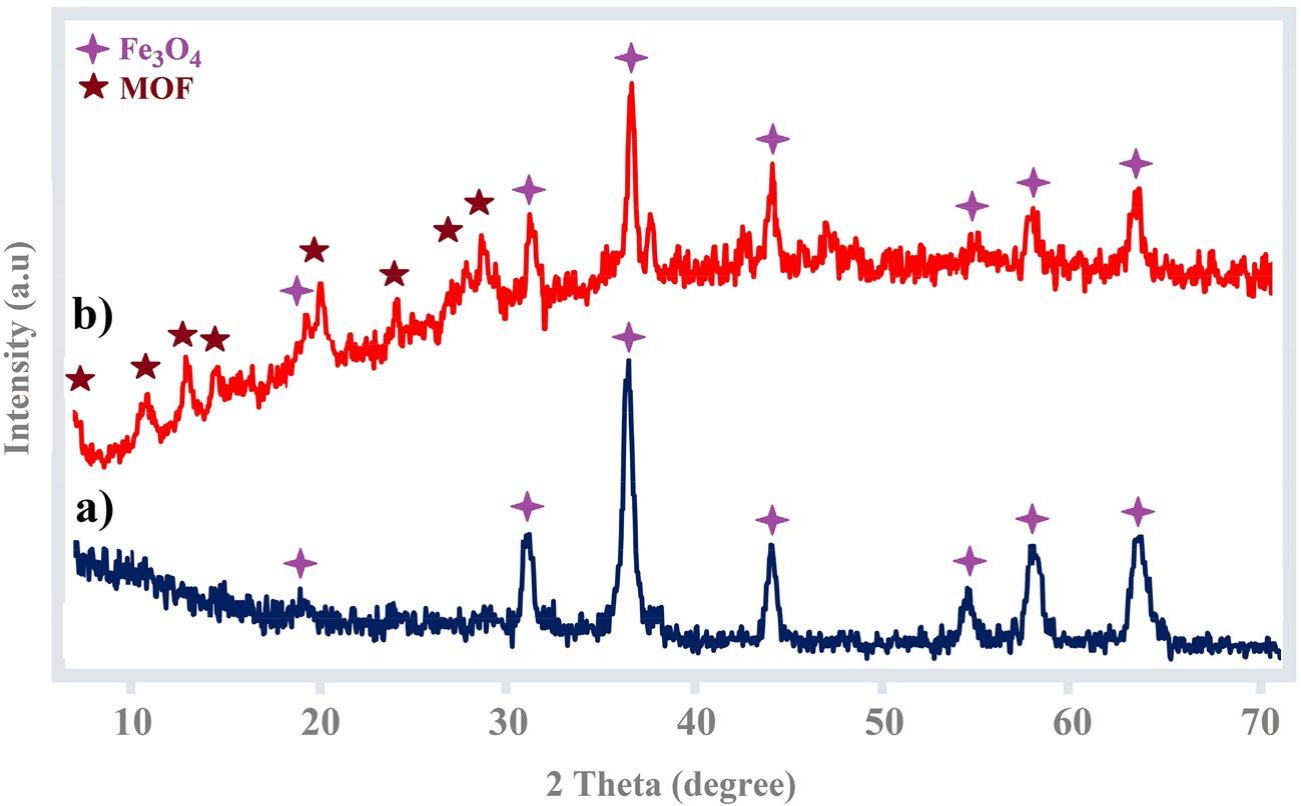
XRD pattern of (**a**) Fe_3_O_4_ and (**b**) Fe_3_O_4_@HKUST-1.

The powder X-ray diffraction (PXRD) analysis was studied for both bare Fe_3_O_4_ and core–shell Fe_3_O_4_@HKUST-1 MNPs (Fig. 8). The PXRD pattern of Fe_3_O_4_ MNPs (Fig. 8a) shows the crystalline phase characteristics with assignments such as (2 2 0), (3 1 1), (4 0 0), (4 2 2), (5 1 1) and (4 4 0) at 2*θ* = 30.10, 35.62, 43.31, 53.30, 57.11 and 62.10 (JCPDS card No. 19–0629)^58^. In the second PXRD pattern of Fe_3_O_4_@HKUST-1 MNPs (Fig. 8b), in addition to Fe_3_O_4_ MNPs peaks, some new peaks are observed between ranges of 2ϴ = 5 to 30 which are attributed to the HKUST-1 MOF shell structures^57^. These observations confirm the successful formation of magnetic MOF structures.

**Figure 9:**
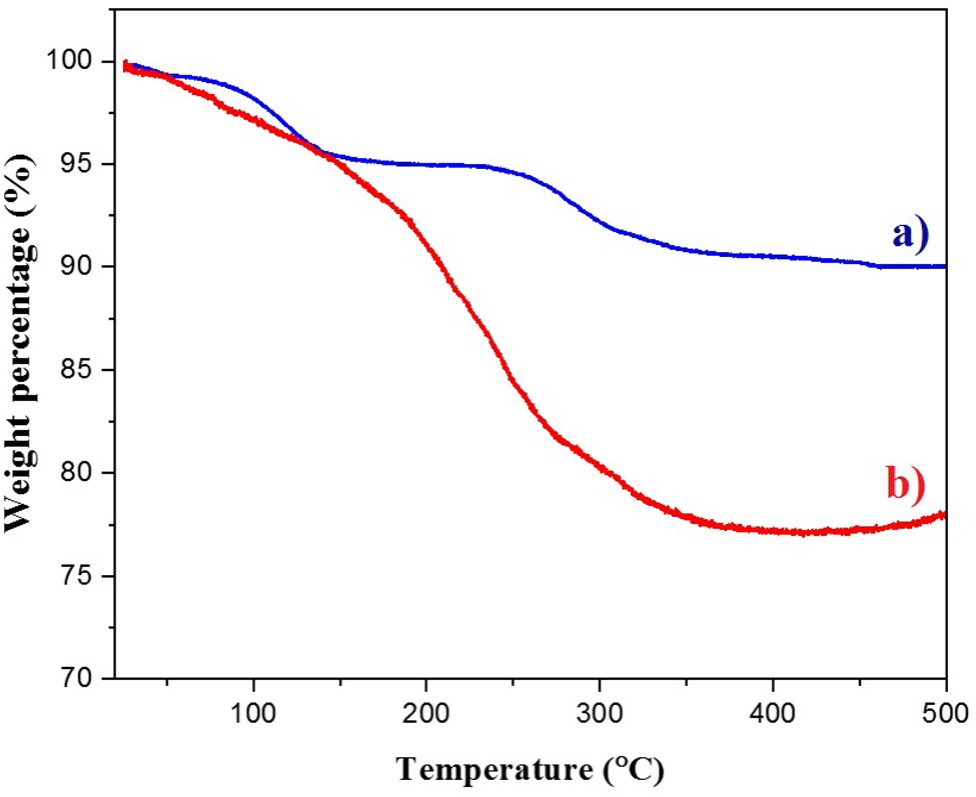
TGA graph of catalyst (**a**) Fe_3_O_4_ and (**b**) Fe_3_O_4_@HKUST-1.

It is known that the TGA analysis is a significant factor to confirm the core–shell structures and evaluate the thermal stability of the samples (Fig. 9). As shown in Fig. 9a, the Fe_3_O_4_ MNPs have high thermal stability due to the iron oxide structures and only 9.958% of sample’s weight has been decreased after increase the temperature to 500 °C. It can be attributed to the decomposition of hydroxyl functional groups on the surface of MNPs or remove of some waters or organic solvents remained in the sample. Interestingly, the TGA graph of Fe_3_O_4_@HKUST-1 MNPs showed more reduction in the weight (about 22.176%, Fig. 9b). The difference between this weigh loss with previous one is about 12% that can be assigned to the decomposition of shell structures^59^.

**Figure 10:**
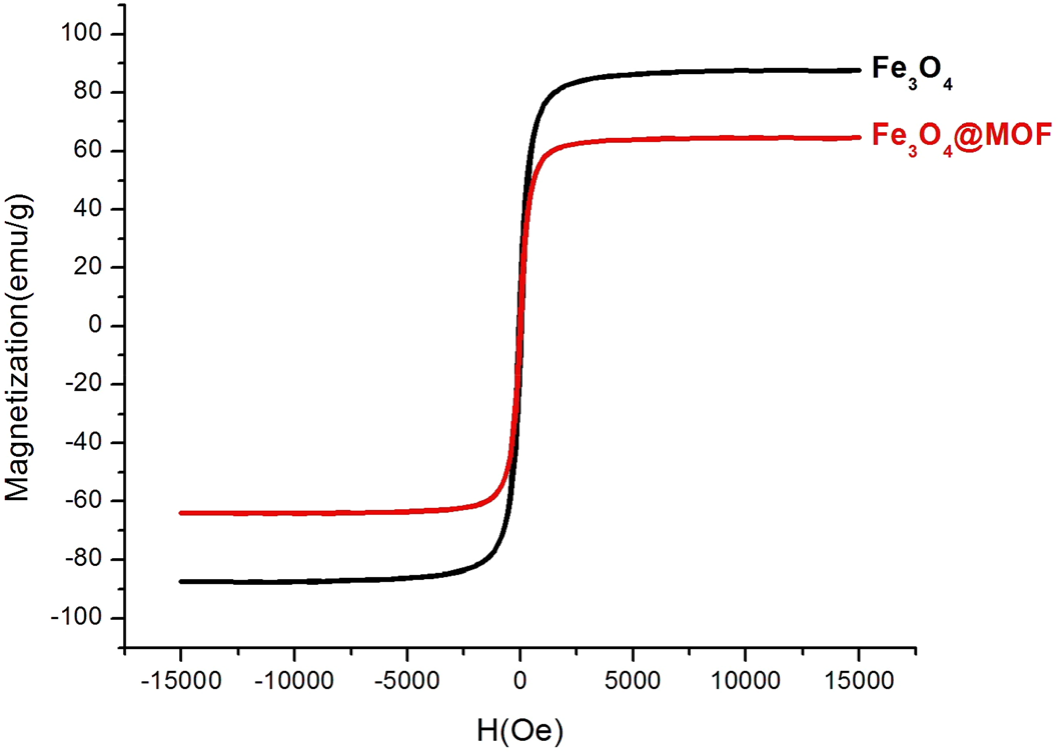
VSM graphs of (**a**) Fe_3_O_4_ and (**b**) Fe_3_O_4_@HKUST-1.

Figure 10 shows magnetic properties of the Fe_3_O_4_ and core–shell MNP structures at room temperature in the range of + 15,000 to −15,000 Oe. Thus, the VSM plot of Fe_3_O_4_ MNPs exhibited the superparamagnetic properties with high saturation magnetization about 85 emu g^−1^ (Fig. 10, black graph). It is noteworthy, this magnetic property was reduced to about 65 emu g^−1^ after coating the MNPs with MOF shell structures (Fig. 10, red graph)^60^. These outcomes approve the successful synthesis of catalyst and it is in agreement with TEM, XRD and TGA results.

It is essential to obtain the amount of copper in the samples. Thereby, its amount in both fresh and reused catalyst (after five times reuse) has been estimated by ICP analysis. According to the ICP results, the weight percentage of Cu in the fresh and reused catalyst were 3.512 and 3.497 wt%, respectively. This analysis strongly confirmed the presence of copper in MOF structures (in accordance with EDX and SEM mapping observations) and also shows the leaching of Cu and shell structures is negligible amount that can be ignored.

After complete characterization the catalyst structure, the catalyst efficiency has been investigated for the synthesis of 1,2,3-triazole derivatives from the three component condensation reaction of different alkyl halides, various phenylacetylene and sodium azide. To this end, initially, the reaction of benzyl bromide, phenylacetylene and sodium azide has been selected as a model reaction by taking into account several important factors such as amount of catalyst, effect of solvent and temperature (Table 1). Hence, at first step, the reaction was carried out without the catalyst under the solvent-free conditions or water as a green solvent (entries 1 and 2). As expected, with solvent-free conditions and without the catalyst, the reaction did not have any desirable product (entry 1) and the amount of product was trace in water (entry 2). With adding the catalyst to the reaction mixture, the yield of the reaction has been increased gradually (entries 3–6). The best amount of catalyst was obtained 30 mg (1.8 mol%) (entry 5). After find the optimum amount of the catalyst, the reaction was examined in the presence of different solvents that such as THF, DMF, MeCN, MeOH, EtOH, H_2_O and solvent-free conditions (entries 7–12). The highest and lowest amount of 1,2,3-triazole compound were obtained in the presence of water and solvent-free condition, respectively (entries 5 and 12). The last step was study the effect of temperature on model reaction (entries 13–15). Therefore, the model reaction was carried out at different temperatures like 25, 50, 100 and 120 °C. The best temperature was 100 °C (reflux condition) and lower and higher temperature was not satisfying. So, the best condition was the using of water as a green solvent under reflux condition and 30 mg (1.8 mol%) of catalyst (entry 5).

**Table 1:** Screening of the reaction conditions for the synthesis of **4a**^a^.

Entry	Conditions	Catalyst (mg, mol%)	Temp. (°C)	Time (min)	TON/TOF^b^	Yield (%)^c^
1	Solvent-free	–	rt	360	–	–
2	H_2_O	–	Reflux	300	–	Trace
3	H_2_O	10, 0.6	Reflux	180	71.7/0.40	43
4	H_2_O	20, 1.2	Reflux	180	56.7/0.31	68
5	H_2_O	30, 1.8	Reflux	120	51.1/0.43	92
6	H_2_O	40, 2.4	Reflux	120	37.5/0.31	90
7	THF	30, 1.8	Reflux	130	18.9/0.14	34
8	DMF	30, 1.8	100	130	23.3/0.18	50
9	MeCN	30, 1.8	Reflux	130	24.4/0.19	44
10	MeOH	30, 1.8	Reflux	130	30.5/0.23	63
11	EtOH	30, 1.8	Reflux	130	33.9/0.26	70
12	Solvent-free	30, 1.8	100	130	–	Trace
13	H_2_O	30, 1.8	r.t	130	–	Trace
14	H_2_O	30, 1.8	50	130	28.3/0.22	51
15	H_2_O	30, 1.8	120	130	50.0/0.38	90
16	H_2_O^d^	30, 1.8	Reflux	120	48.3/0.40	87
17	H_2_O^e^	30, 1.8	Reflux	120	46.1/0.38	83
18	H_2_O^f^	30, 1.8	Reflux	120	44.4/0.37	80

^a^Reaction condition: Benzyl bromide (1 mmol), phenylacetylene (1 mmol), sodium azide (1.2 mmol).

^b^Turn Over Number (yield of the reaction/mol%)/Turn Over Frequency (TON/time of the reaction).

^c^Based on isolated yield.

^d^In the presence of HKUST-1.

^e^In the presence of Cu(OAc)_2_.

^f^In the presence of CuCl_2_.

In the second step and after finding the optimized reaction condition, the generality of this research was investigated in the reaction of different alkyl and benzyl halides with sodium azide and various phenyl acetylene and also in the attendance of 30 mg (1.8 mol%) of Fe_3_O_4_@HKUST-1 catalyst and water as a green solvent at 100 °C (Table 2). It is worth mentioning that, benzyl halides (entries 1–8) reacted better compared to alkyl halides (entries 9–11) on the basis of yield of the reaction and gave the product with highest amounts of yield. On the other hand, the phenylacetylene derivatives with no substituent (entries 1, 5, 6, and 8) or with electron-withdrawing group (entry 13) have better yield of the reaction compared to electron-donating groups (entries 12, 14 and 15). After that, in order to investigate the catalyst efficiency, the effects of bare MOF (HKUST-1) without the core (Fe_3_O_4_) (entry 16) and some Cu salts like Cu(OAc)_2_ and CuCl_2_ (entries 17 and 18) have been tested in the model reaction under optimized conditions. As is evident, the yields of the reaction in presence of HKUST-1, copper acetate and copper chloride were 87, 83 and 80% respectively that they are a little bit lower than Fe_3_O_4_@HKUST-1 which can be attributed to the nano structures of the final catalyst.

**Table 2:**
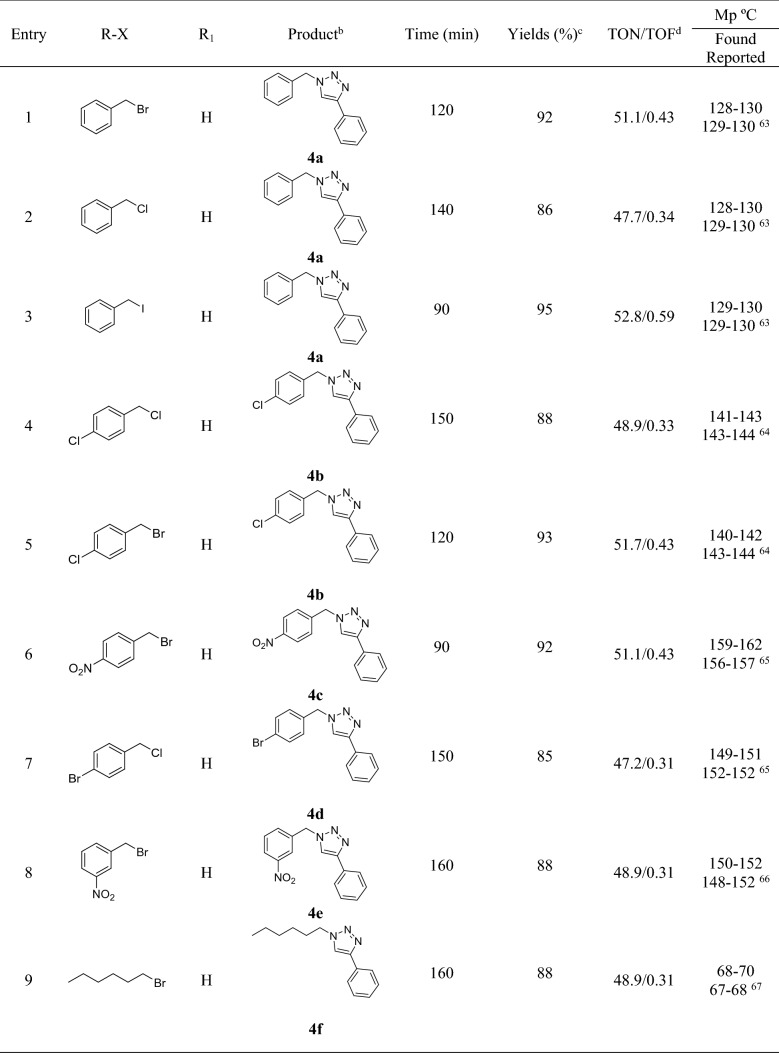

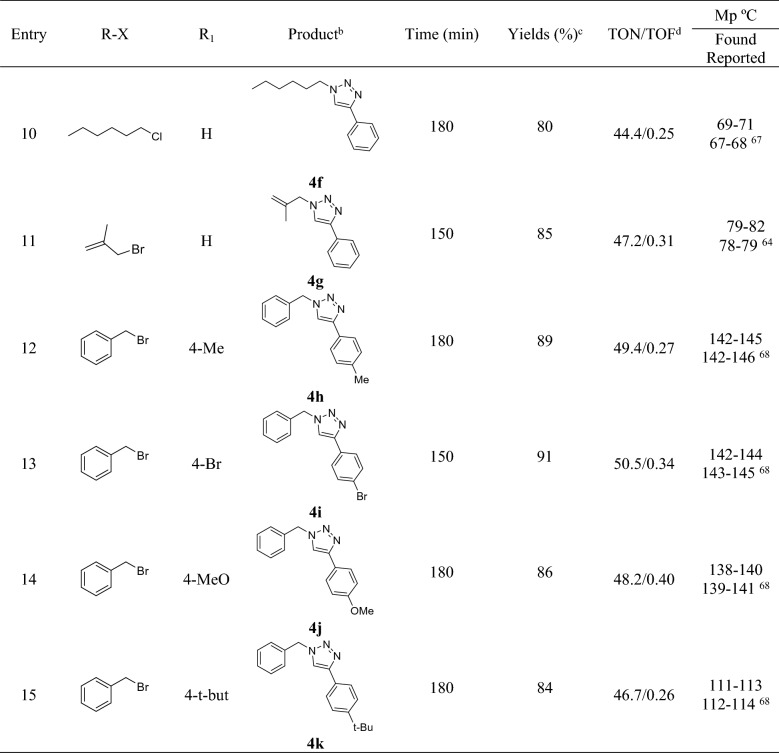
Synthesis of 1,2,3-triazole derivetives using Fe_3_O_4_@HKUST-1 nanocatalyst^a^.

^a^Reaction condition: Benzyl/alkyl halide (1.0 mmol), phenylacetylene derivatives (1.0 mmol), sodium azide (1.2 mmol), catalyst (30 mg, 1.8 mol%) and H_2_O at 100 °C.

^b^All the products were charchetrized by ^1^H NMR, ^13^C NMR and FT-IR.

^c^Isolated Yields.

^d^Turn Over Number (yield of the reaction/mol%)/Turn Over Frequency (TON/time of the reaction).

**Figure 11:**
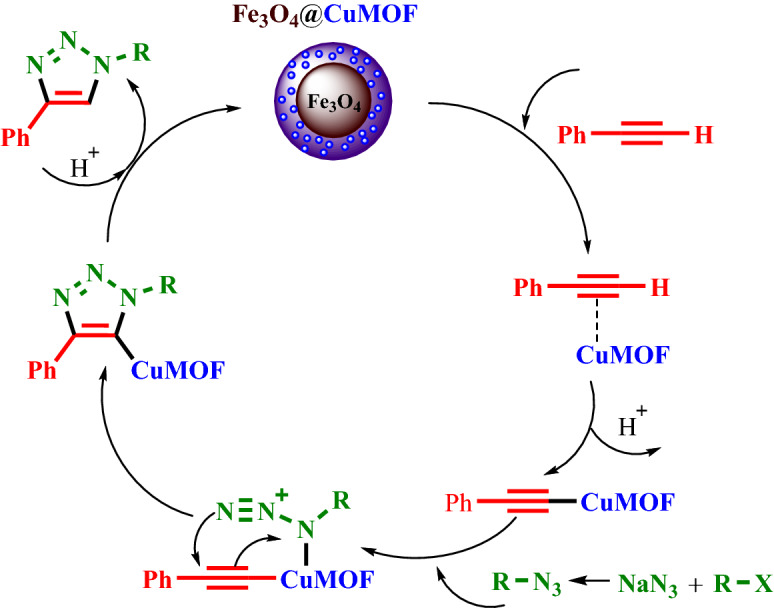
The proposed mechanism for the synthesis of 1,2,3-triazoles in the presence of Fe_3_O_4_@HKUST-1 Cu catalyst.

According to the literature^61,62^, a suggested reaction mechanism for the 1,3-dipolar cycloaddition reaction in the attendance of Fe_3_O_4_@HKUST-1 catalyst was proposed (Fig. 11). As mentioned before in the shell structure, the copper (II) was existed that it can be acted as a Lewis acid and catalyzed the synthesis of 1,2,3-triazole derivatives properly. On this basis, in the first step, the phenyl acetylene can be coordinated to Cu centers in the MOF structures and the Cu–alkylidine intermediate is formed. Next, the alkyl or benzyl halide as an electrophile can react with sodium azide as nucleophile and the alkyl or benzyl azide are produced. Afterwards, it attacks with previous formed Cu intermediate and finally, after simple intramolecular cyclization, the final product is generated.

**Figure 12:**
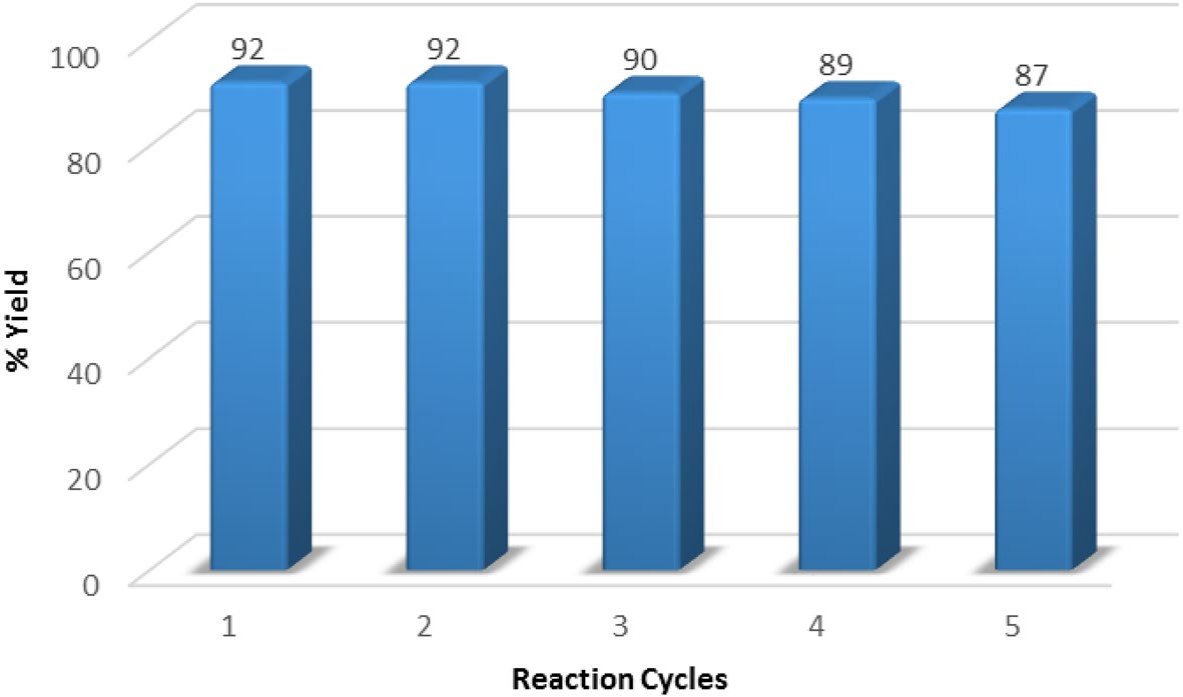
Reusability of catalyst for model reaction.

Our catalyst exhibited high catalytic stability in repeated model reaction. On this basis, after finalization the model reaction, the Fe_3_O_4_@HKUST-1 catalyst was separated from the reaction mixture by an external magnet and washed several times with DMF and acetone, dried in vacuum oven at 50 °C for 12 h and reused in the next model reaction. It can catalyze the synthesis of 1,2,3-triazole derivatives with high isolated yield and after five time reusing the catalyst, the yield of the reaction decreased very low that it can be ignored (Fig. 12). These observations showed the structure of catalyst and especially shell structures stay without change during the reaction and probably no leaching of Cu and MOF materials has been occurred during the reaction.

**Figure 13:**
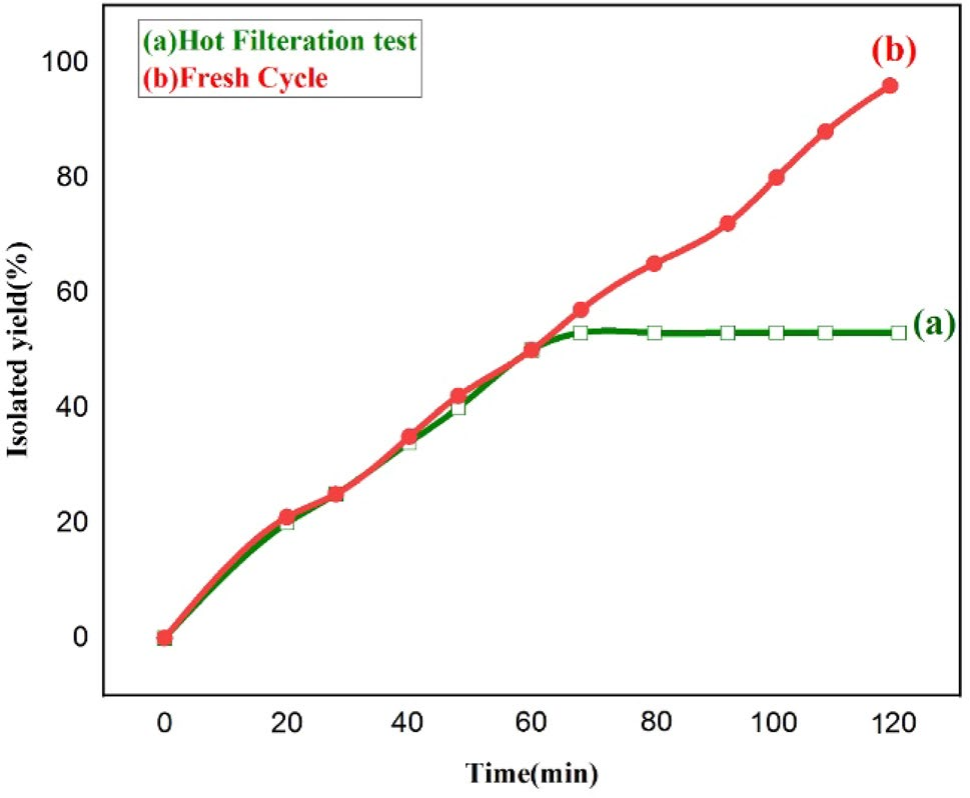
Time-dependent correlation of the product yield in hot filtration test.

Hot filtration test was also performed to scrutiny the stability of catalyst. In this regard, the model reaction of benzyl bromide (1.0 mmol), sodium azide (1.2 mmol) and phenyacetylene (1.0 mmol) has been carried out in the presence of optimum amount of catalyst (1.8 mol%) and water under reflux condition for 60 min. After progress the reaction for approximately 50%, the Fe_3_O_4_@HKUST-1 catalyst has been separated from the reaction mixture with an external magnet and then, the reaction performed for another 60 min. In interesting notice, without the catalyst, the reaction was not carried out that it was monitored by TLC (Fig. 13). A good agreement with ICP results and shows that no leaching of Cu or MOF shell structures have been happened during the 1,3-dipolar cycloaddition reaction. Also, to elucidate the stability of catalyst, after five cycles in the model reaction, any structural changes of catalyst were studied by FT-IR, SEM and ICP techniques. It is clearly evident from the FT-IR spectrum of the 5th reused catalyst that no significant changes in the frequencies, intensities and shapes of absorption bands were observed (Fig. S1). Moreover, the morphology and size of 5th reused catalyst has been investigated bey SEM technique (Fig. S2). The SEM image of resued catalyst (Fig. S2b) was aproximately similar with the SEM image of fersh catalyst (Fig. S2a) and there are not any significant differences in size and morphlogy. Besides, as mentioned in previous sections, the ICP-OES analysis showed that the amount of Cu in reused catalyst is 3.497 wt% that the decrease is very low and it can be ignored. Based on this valuable obtained data, no significant leaching of MOF was obsedrved from the surface of Fe_3_O_4_ NPs.

To show the merit of present study compared to previously reported ones by using copper-based catalyst, the effect of our magnetic CuMOF catalyst has been compared with other Cu catalysts in the reaction of benzyl bromide, sodium azide and phenylacetylene (Table 3). Up to now, different homogeneous and heterogeneous Cu catalysts have been used for the azide-alkyne cycloaddition reaction (entries 1–8), but some of them suffer from long reaction time (entries 1–5), using hazardous solvent and reaction conditions (entries 1,3,4 and 7) and more importantly problems to reuse the catalyst (entries 1–3, and 5). Due to the heterogeneous nature and magnetic properties of our catalyst, it can be separated from the reaction mixture by a simple external magnet and it can catalyze the 1,3-diplar cycloaddition reaction in shorter reaction time and greener condition (use water, entry 9).

**Table 3:** Comparison of Fe_3_O_4_@HKUST-1 catalyst with other copper catalysts for the synthesis of 1,2,3-triazole **4a.**

Entry	Catalyst	Solvent	Temp. (oC)	Time (h)	Yield (%)^a^	Refs.
1	Nano CuO	H_2_O/THF	60	24	95	^43^
2	Graphite-Cu NPs	H2O/MeOH	70	12	91	^69^
3	GO-Metformin-Cu	H_2_O	rt	0.5	96	^70^
4	Cu polymer-Fe_3_O_4_	H_2_O	50	2.0	96	^71^
5	Cu-MWCNT@Fe_3_O_4_	H_2_O	50	1.0	99	^72^
6	CuII–Hydrotalcite	MeCN	rt	3.0	86	^73^
7	Cu/SiO_2_	THF	rt	2.0	99	^74^
8	Thiourea CuCl	Solvent-free	rt	2.0	96	^75^
9	Fe_3_O_4_@MOF	H_2_O	100	2.0	92	This study

^a^Based on isolated yield.

## Conclusion

In summary, a new magnetic MOF catalyst based on copper has been described for the synthesis of 1,2,3-triazole derivatives via the three component condensation reaction of various benzyl/alkyl halides, sodium azide and different phenyl acetylene derivatives. However, this reaction has been carried out with various homogeneous and heterogeneous Cu catalysts and also this reaction is famed to Cu azide-alkyne cycloaddition reaction, but this study is the first report for the use of magnetic CuMOF in the azide-alkyne cycloaddition reaction. Surprisingly, this catalyst can catalyze the synthesis of 1,2,3-triazole derivatives in short reaction time, good to excellent yields and more importantly in the presence of water as a green and environmentally friendly solvent.

## Experimental

### General

All the materials and reagents were bought from Merck and Sigma-Aldrich companies without any further purification. NMR spectra were obtained with a Bruker AC instrument at 300 MHz in DMSO‐d6. Mass spectra were obtained with a Varian Mat CH‐7 at 70 eV. FT‐IR spectra were recorded with a Nicolet Avatar 370 FT‐IR spectrometer. TGA was performed with a Shimadzu thermogravimetric analyzer (TG‐ 50) under air atmosphere at a heating rate of 10 °C min^−1^. The crystal structure of the catalyst was analyzed using XRD with a Bruker D8 ADVANCE diffractometer using a Cu target (*λ* = 1.54 Å). TEM was performed with a Leo 912AB (120 kV) microscope (Zeiss, Germany). ICP analysis was carried out with a Varian VISTA‐ PRO, CCD (Australia). Elemental compositions were determined with EDX analysis (model 7353, Oxford Instruments, UK), with 133 eV resolute ion. The magnetic property of the catalyst was measured using VSM (model 7400, Lake Shore). Melting points of products were recorded with an Electrothermal type 9200 melting point apparatus.

### Preparation of catalyst (Fe_3_O_4_@HKUST-1)

The catalyst has been prepared according to the literature^55,56^. At the outset, the Fe_3_O_4_ MNPs have been prepared by using FeCl_3_ and hydrothermal process^55^, and then, the surface of these MNPs has been modified by mercaptoacetic acid and finally, these modified MNPs have been coated with HKUST-1 MOF by a versatile layer-by-layer assembly method and using copper acetate and 1,3,5-benzenetricarboxylic acid (H3-BTC) (Fig. 1)^56^.

### General procedure for the synthesis of products 4a-k

To a mixture of phenyl acetylene derivatives (1.0 mmol), benzyl/alkyl halide (1.0 mmol) and sodium azide (1.2 mmol), in 10 mL water, 30 mg (1.8 mol%) of Fe_3_O_4_@HKUST-1 catalyst was added and refluxed for the time mentioned in Table 2. The progress of the reaction was monitored by TLC and upon the completion the reaction, the reaction mixture was diluted with ethyl acetoacetate and the Fe_3_O_4_@HKUST-1 catalyst was separated by an external magnet. Next, the organic part was separated from the water and it was dried on anhydrous Na_2_SO_4_ and after solvent evaporation, the final products have been purified by recrystallization from ethanol to obtain the compounds **4a-k**.

## Supplementary Information

Supplementary Information.
