# Supplementary material for: A magnetic copper organic framework material as an efficient and recyclable catalyst for the synthesis of 1,2,3-triazole derivatives
Source: Sci Rep. 2021 Oct 15;11:20514. doi: 10.1038/s41598-021-00012-3 (PMC8519936; doi:10.1038/s41598-021-00012-3)
Supplement: Supplementary file 1 — Supplementary Information. [file 41598_2021_12_MOESM1_ESM.docx]

**Supporting Information**

[**A magnetic copper organic framework material as an efficient and recyclable catalyst for**](https://www.sciencedirect.com/science/article/pii/S0021951720301482?casa_token=DXGk42I4FkQAAAAA:zDuRKFH9QmP1yfgM5_cnvxgy1QqwZcgZs9qIApWQjIlj_4-yS9wufRtZ5HVKt3xKue1cqL_pNUg) **the synthesis of 1,2,3-triazole derivatives**

Elham Arefi, Amir Khojastehnezhad, and Ali Shiri*

*Department of Chemistry, Faculty of Science, Ferdowsi University of Mashhad, Mashhad, Iran*

*E-mail: alishiri@um.ac.ir*

**
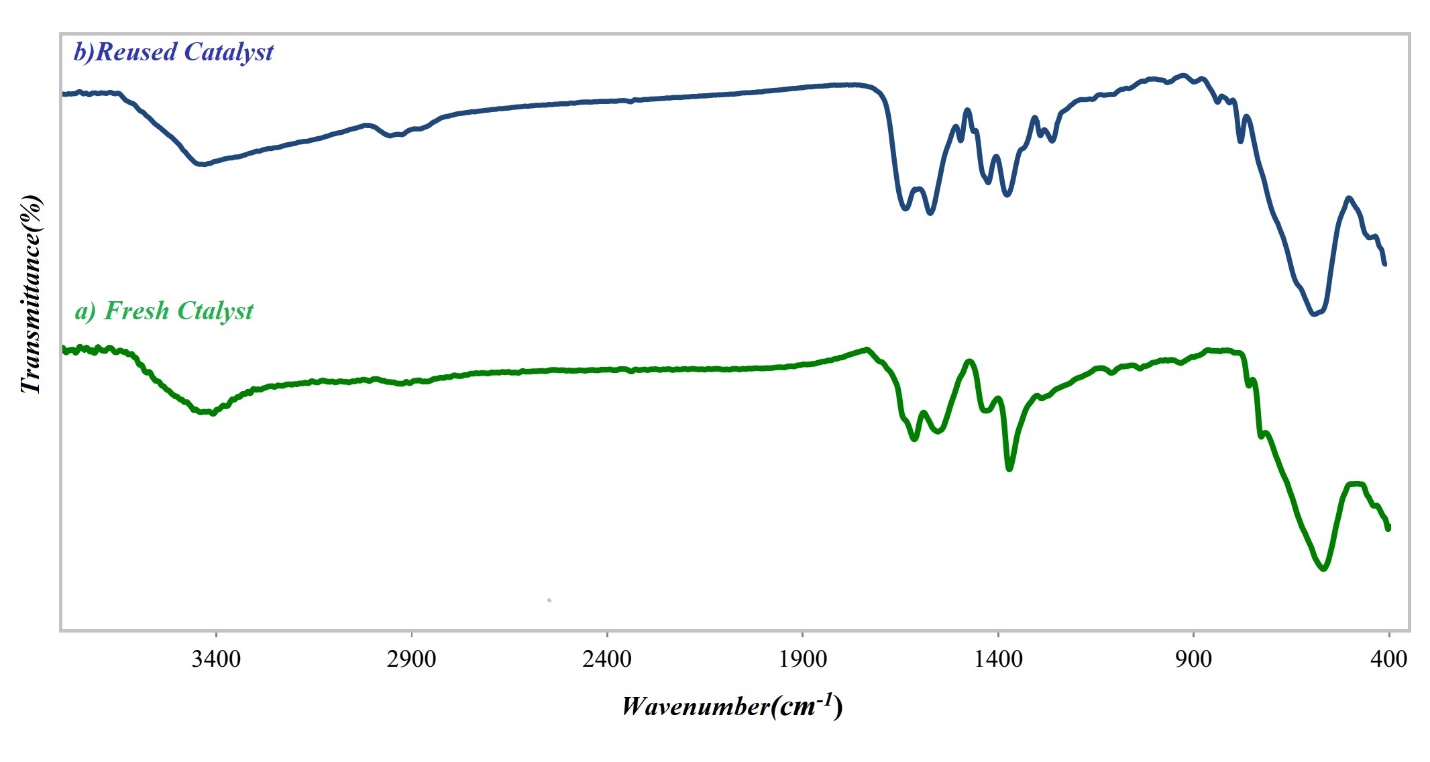
**

Fig. S1. (a) FT-IR of fresh Fe_3_O_4_@HKUST-1 and (b) reused Fe_3_O_4_@HKUST-1

**
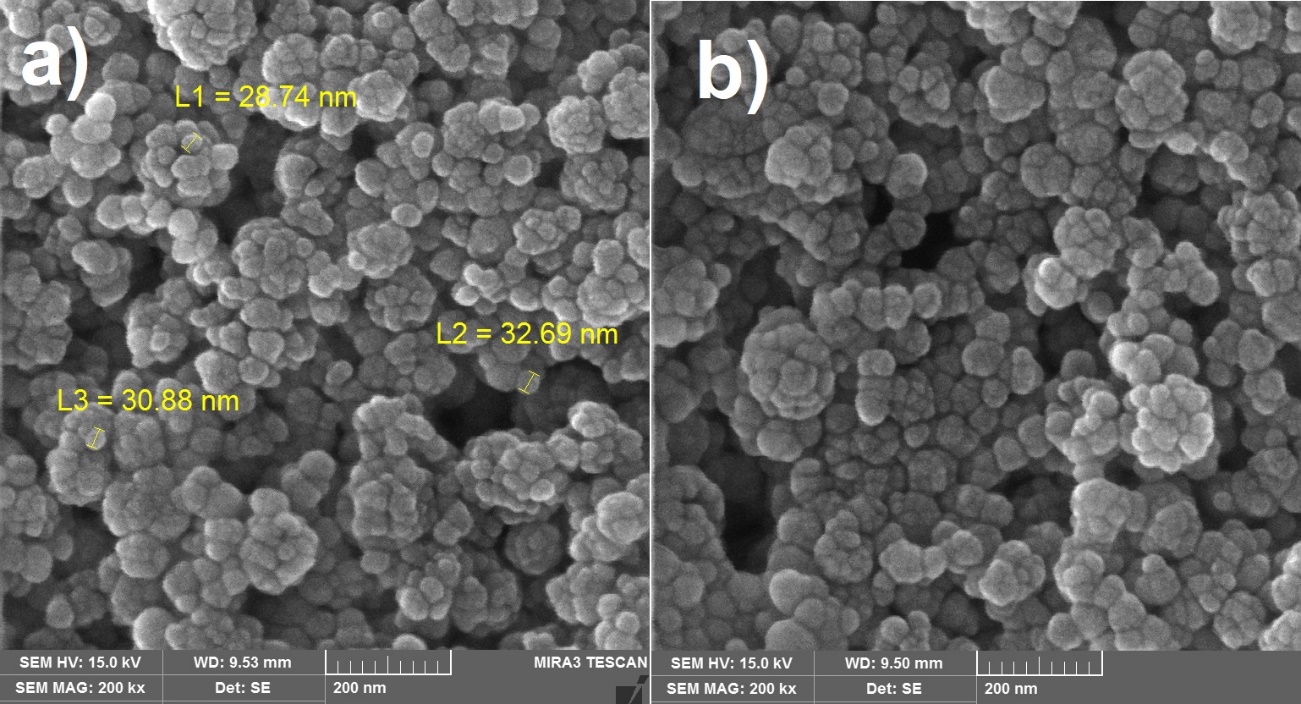
**

Fig. S2. (a) TEM images of fresh Fe_3_O_4_@HKUST-1 and (b) reused Fe_3_O_4_@HKUST-1

1-benzyl-4-phenyl-1H-1,2,3-triazole (Table 2, compound 4a)

M.p. 128-130 ^o^C; IR (KBr): 3145, 1468, 1361, 1223, 1075, 1045, 768 cm^-1^; ^1^H NMR (300 MHz, DMSO-d_6_): *δ* (ppm) = 8.70-8.65 (m, 1H), 7.92-7.89 (m, 2H), 7.49-7.31 (m, 8H), 5.68 (s, 2H); ^13^C NMR (75 MHz, DMSO-d_6_): *δ* (ppm) = 147.2, 136.5, 131.2, 129.4, 129.3, 128.6, 128.4, 125.6, 122.0, 53,5.

1-(4-nitrobenzyl)-4-phenyl-1H-1,2,3-triazole (Table 2, compound 4c)

M.p. 159-162 ^o^C; IR (KBr): 3125, 3080, 1607, 1519, 1350, 1221, 1077, 1044, 763, 732, 690 cm^-1^; ^1^H NMR (300 MHz, DMSO-d_6_): *δ* (ppm) = 8.72 (s, 1H), 8.29-8.26 (d, 2H, *J* = 9.1 Hz), 7.89-7.87 (d, 2H, *J* = 6.2 Hz), 7.61-7.58 (d, 2H, *J* = 9.1 Hz), 7.50-7.35 (m, 3H), 5.87 (s, 2H); ^13^C NMR (75 MHz, DMSO-d_6_): *δ* (ppm) = 147.7, 147.3, 143.9, 131.0, 129.5, 129.4, 128.5, 125.7, 124.4, 122.5, 52,6.

1-benzyl-4-p-tolyl-1,2,3-triazole (Table 2, compound 4h)

M.p. 142-145 ^o^C; IR (KBr): 3026, 2981, 1496, 1453, 1222, 1163, 1047, 820, 793, 720, 695 cm^1^; ^1^H NMR (300 MHz, DMSO-d_6_): *δ* (ppm) = 7.61-7.7 (m, 3H), 728-7.59 (m, 7H), 5.66 (s, 2H), 2.34 (s, 3H) ^13^C NMR (75 MHz, DMSO-d_6_): *δ* (ppm) = 147.9, 137.6, 136.5, 133.0, 128.7, 128.6, 128.5, 128.3, 125.5, 121.6, 53,4, 21.31.

1-benzyl-4-(4-bromophenyl)-1H-1,2,3-triazole (Table 2, compound 4i)

M.p. 142-144 ^o^C; IR (KBr): 3122, 3076, 2949, 1720, 1449, 1336, 1221, 1098, 1067, 1011, 975, 820, 707, 699 cm^-1^. ^1^H NMR (500 MHz, DMSO-d6) *δ* (ppm) = 5.65 (s, 2H), 7.36 (s, 5H), 7.63 (s, 2H), 7.80 (s, 2H), 8.71 (s, 1H); ^13^C NMR (125 MHz, DMSO-d_6_) *δ* (ppm) = 53.7, 121.5, 122.6, 127.8, 128.6, 128.8, 129.4, 130.6, 132.5, 136.5, 146.2.

1-benzyl-4-(4-methoxyphenyl)-1H-1,2,3-triazole (Table 2, compound 4j)

M.p. 138-140 ^o^C; ^1^H NMR (500 MHz, DMSO-d_6_) *δ* (ppm) = 3.80 (s, 3H), 5.64 (s, 2H), 7.03 (d, *J* = 10 Hz, 2H), 7.36-7.41 (m, 4H), 7.77 (m, 2H), 7.90 (s, 1H), 8.54 (s, 1H), ; ^13^C NMR (125 MHz, DMSO-d_6_) *δ* (ppm) = 53.4, 55.6, 114.7, 121.0, 123.7, 126.9, 129.1, 129.2, 129.3, 136.5, 146.5, 159.4.

**
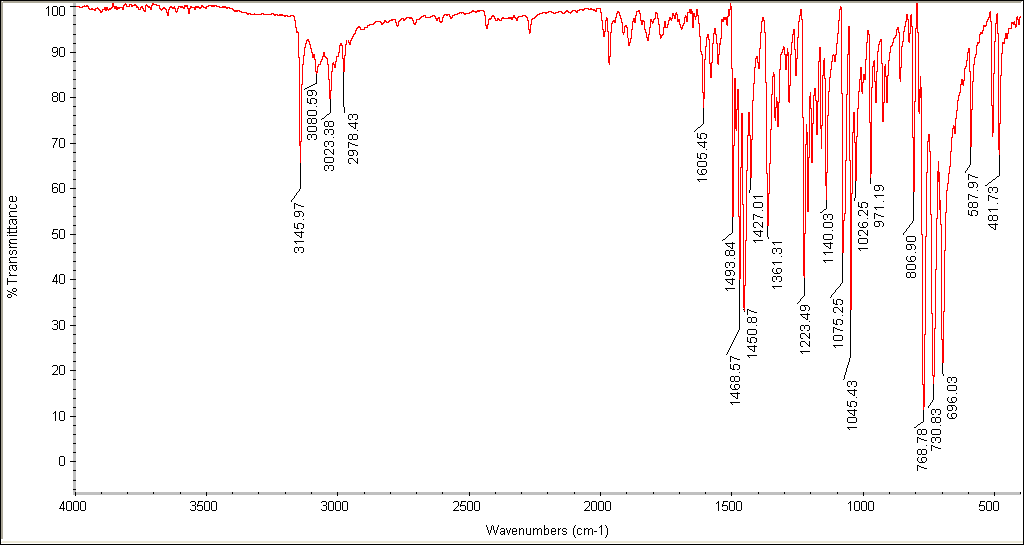
**

Fig. S3. FT-IR of 1-benzyl-4-phenyl-1H-1,2,3-triazole (Table 2, compound 4a)


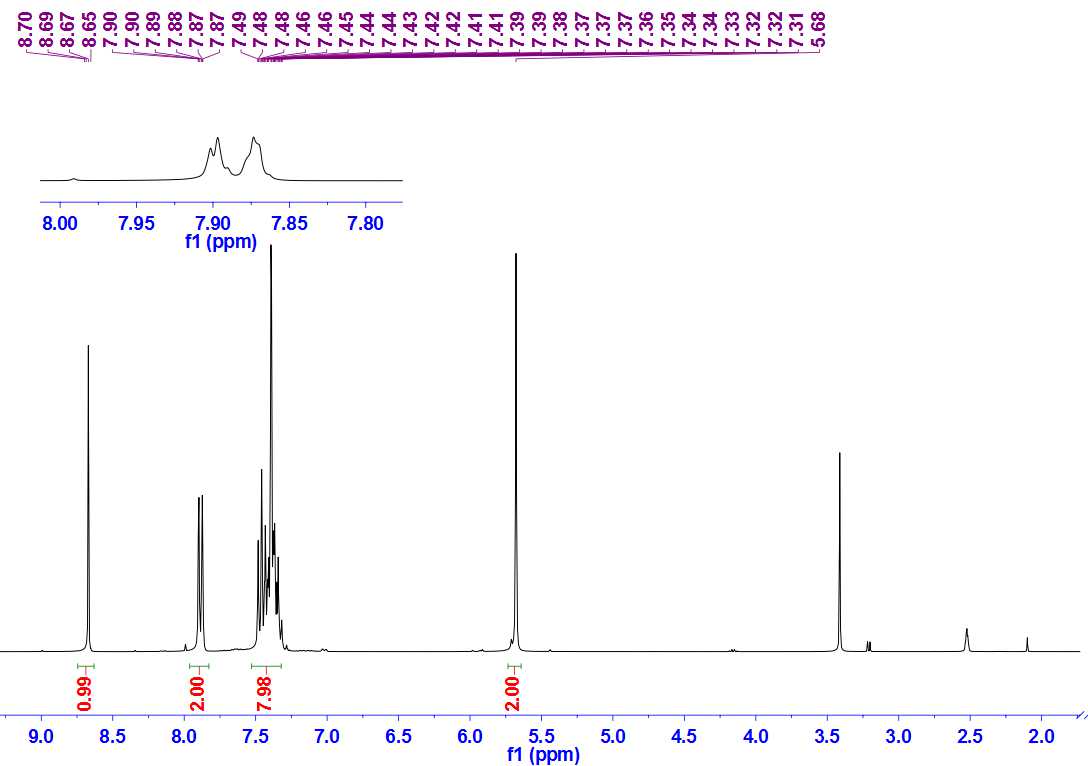


Fig. S4. ^1^H-NMR of 1-benzyl-4-phenyl-1H-1,2,3-triazole (Table 2, compound 4a)


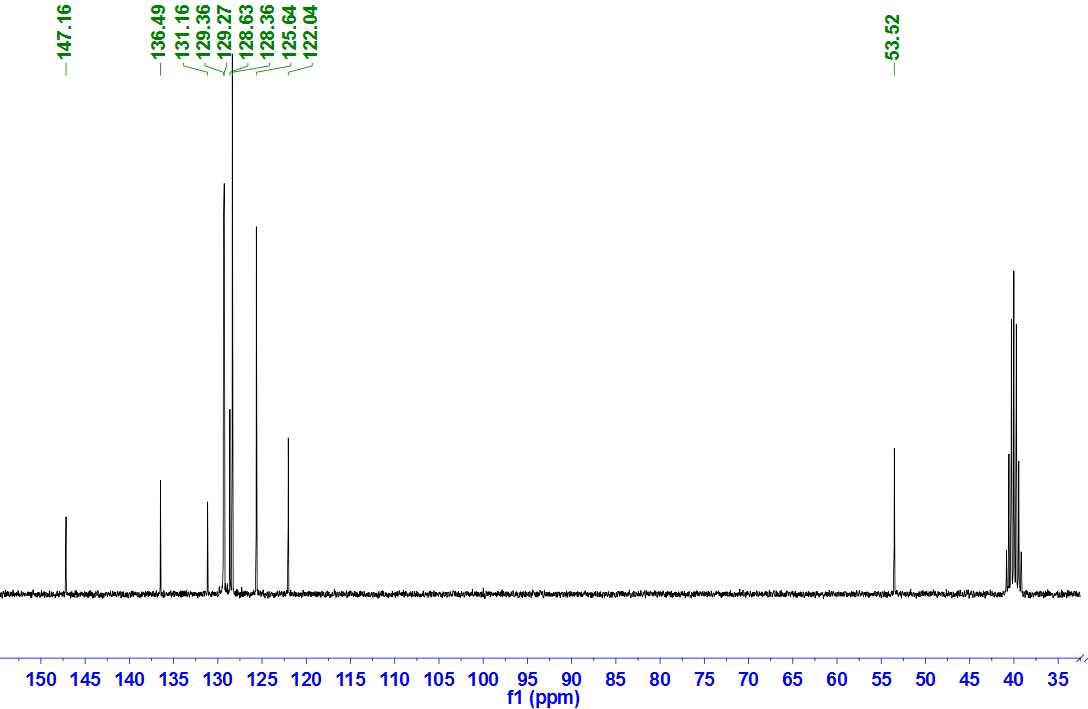


Fig. S5. ^13^C-NMR of 1-benzyl-4-phenyl-1H-1,2,3-triazole (Table 2, compound 4a)


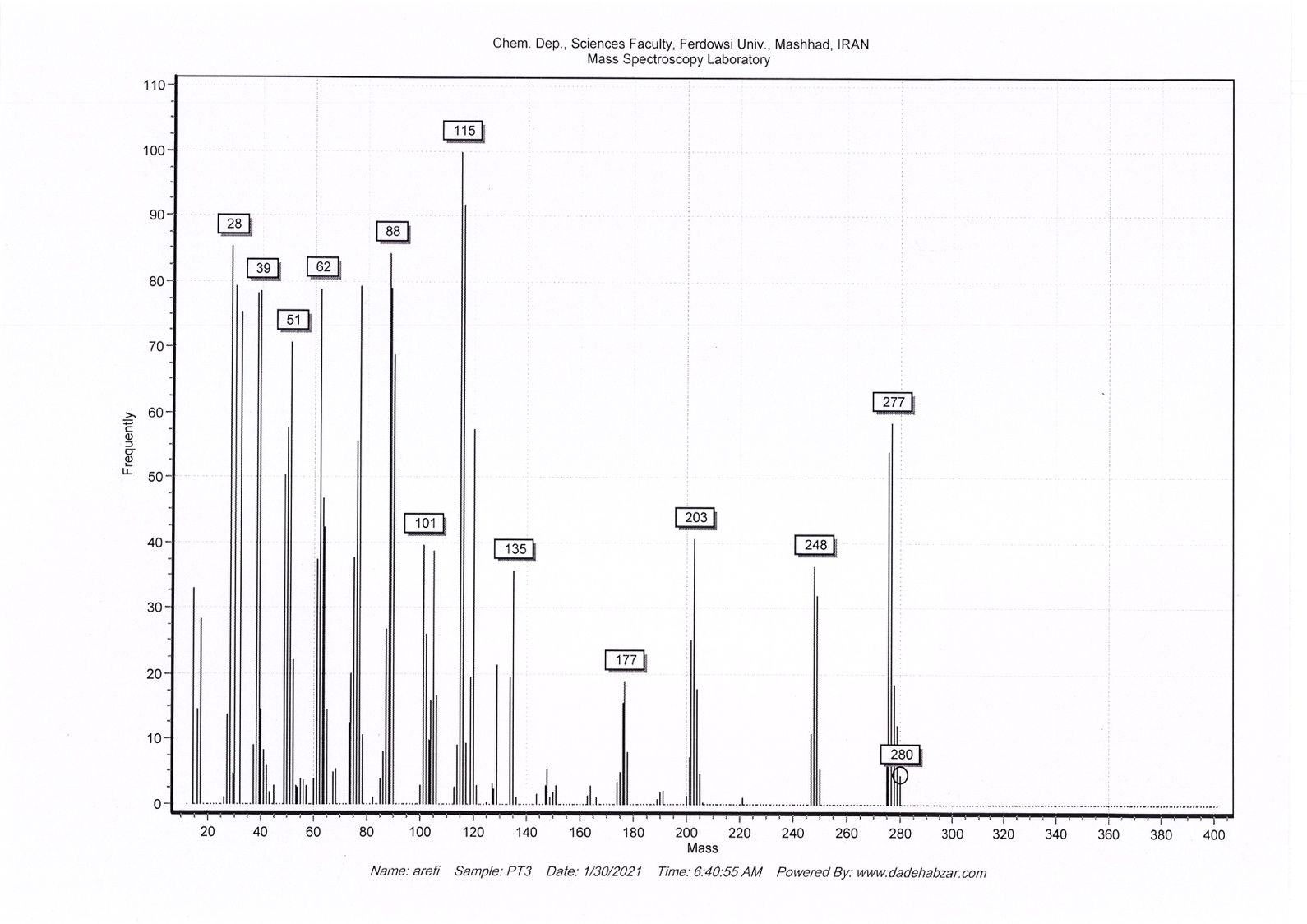


Fig. S6. Mass Spectrum of 1-(4-nitrobenzyl)-4-phenyl-1H-1,2,3-triazole (Table 2, compound 4c)

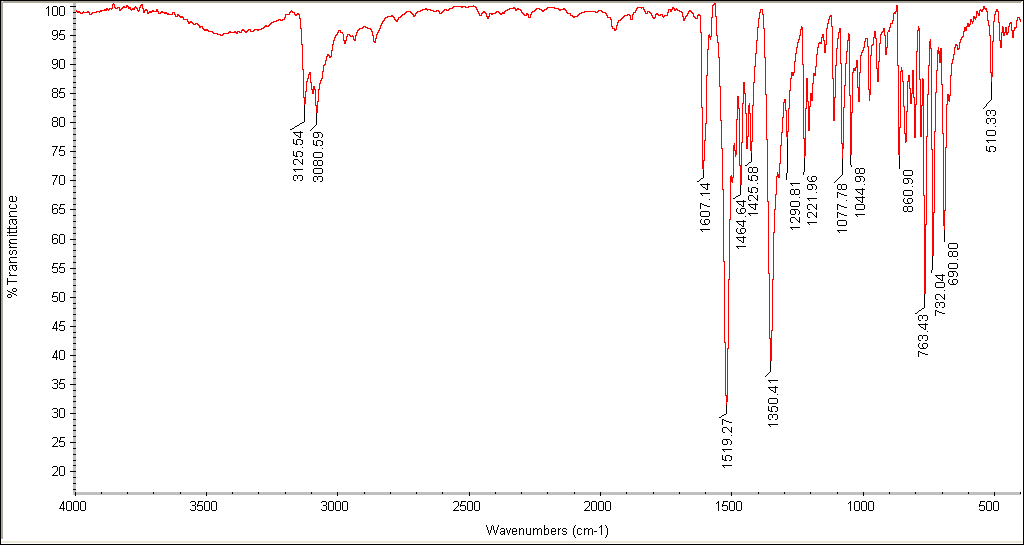


Fig. S7. FT-IR of 1-(4-nitrobenzyl)-4-phenyl-1H-1,2,3-triazole (Table 2, compound 4c)


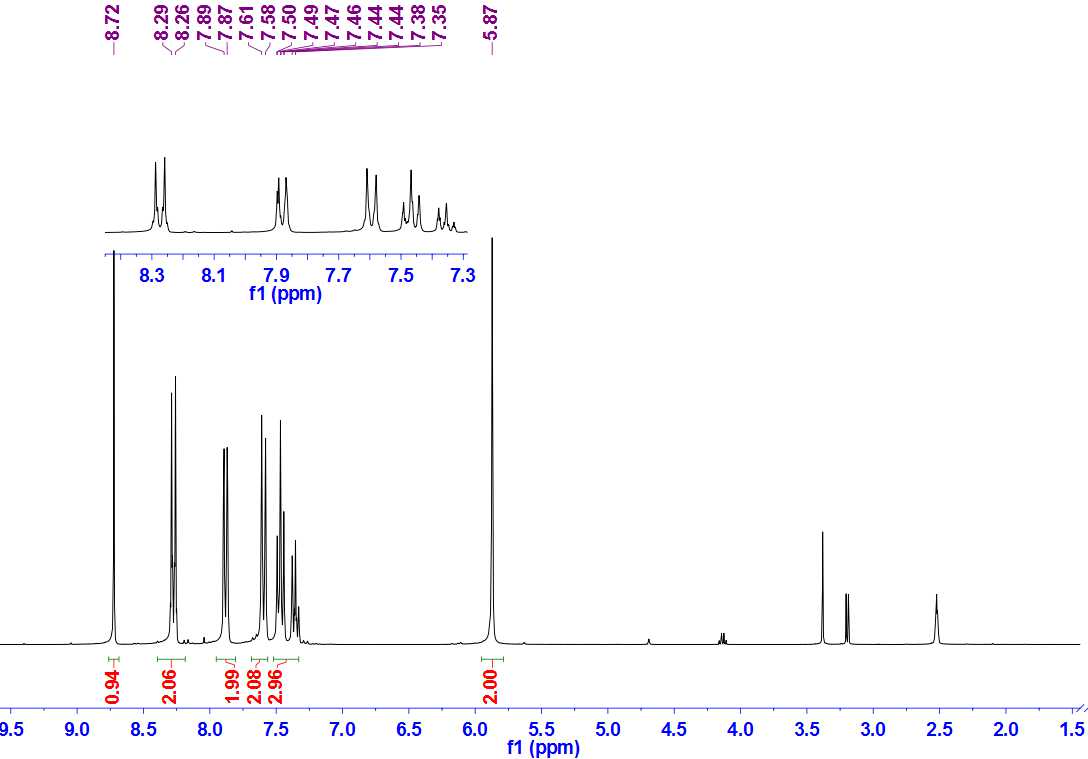


Fig. S8. ^1^H-NMR of 1-(4-nitrobenzyl)-4-phenyl-1H-1,2,3-triazole (Table 2, compound 4c)

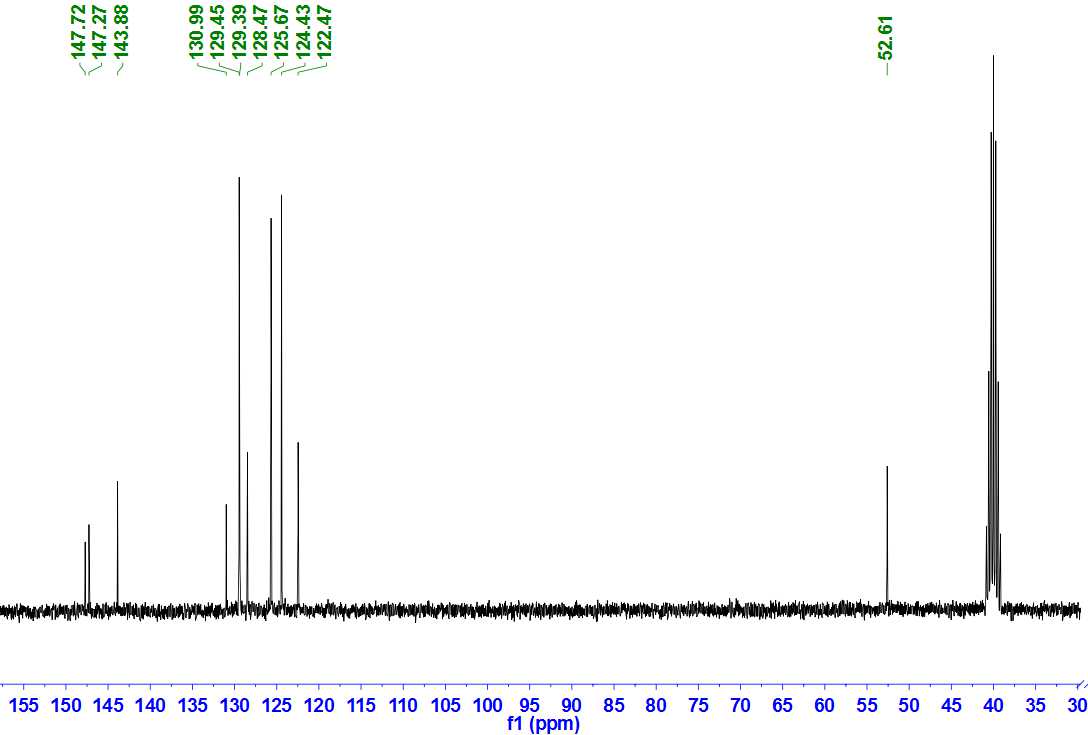


Fig. S9. ^13^C-NMR of 1-(4-nitrobenzyl)-4-phenyl-1H-1,2,3-triazole (Table 2, compound 4c)


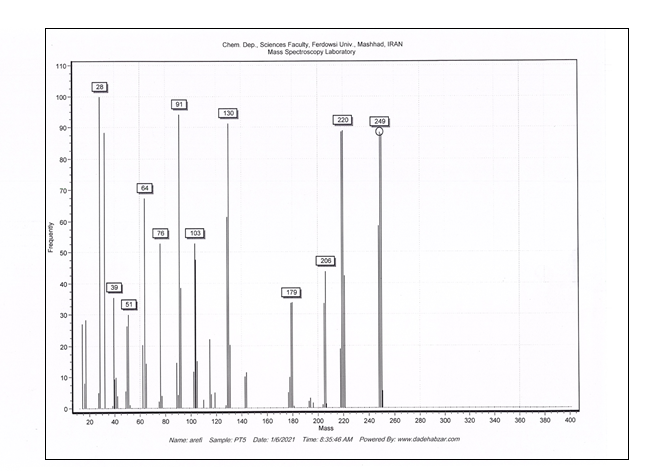


Fig. S10. Mass spectrum of 1-benzyl-4-p-tolyl-1,2,3-triazole (Table 2, compound 4h)

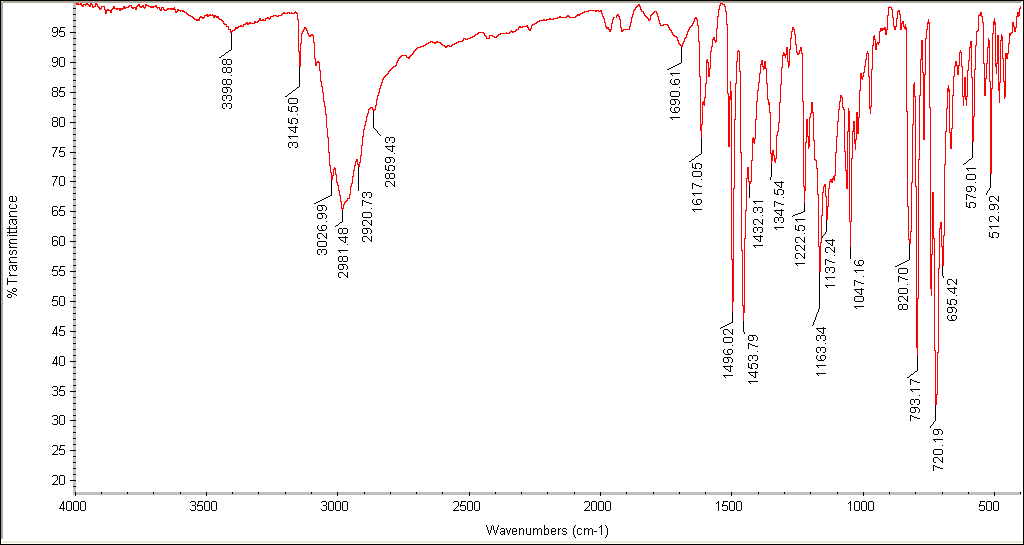


Fig. S11. FT-IR of 1-benzyl-4-p-tolyl-1,2,3-triazole (Table 2, compound 4h)


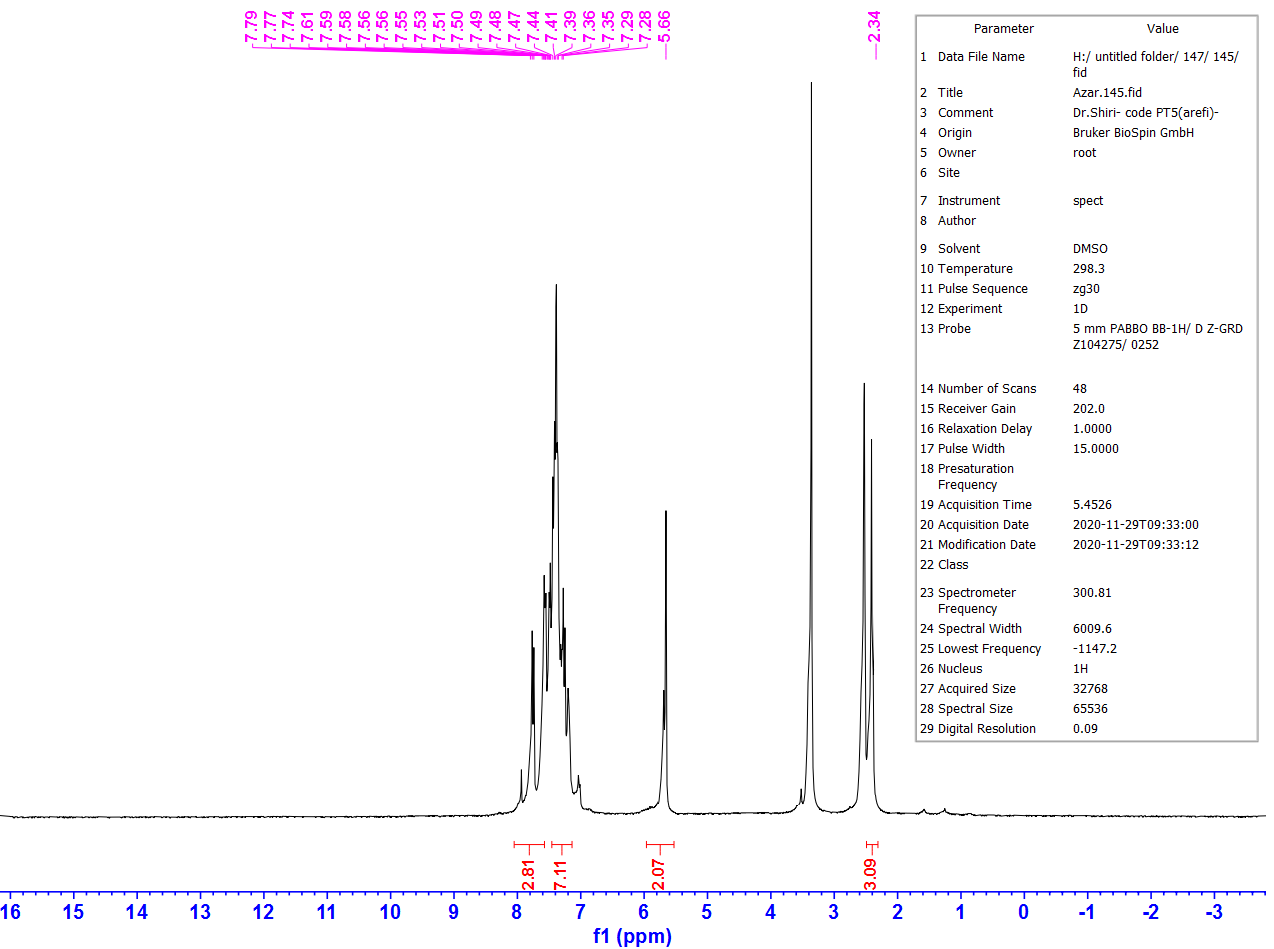


Fig. S12. ^1^HNMR of 1-benzyl-4-p-tolyl-1,2,3-triazole (Table 2, compound 4h)

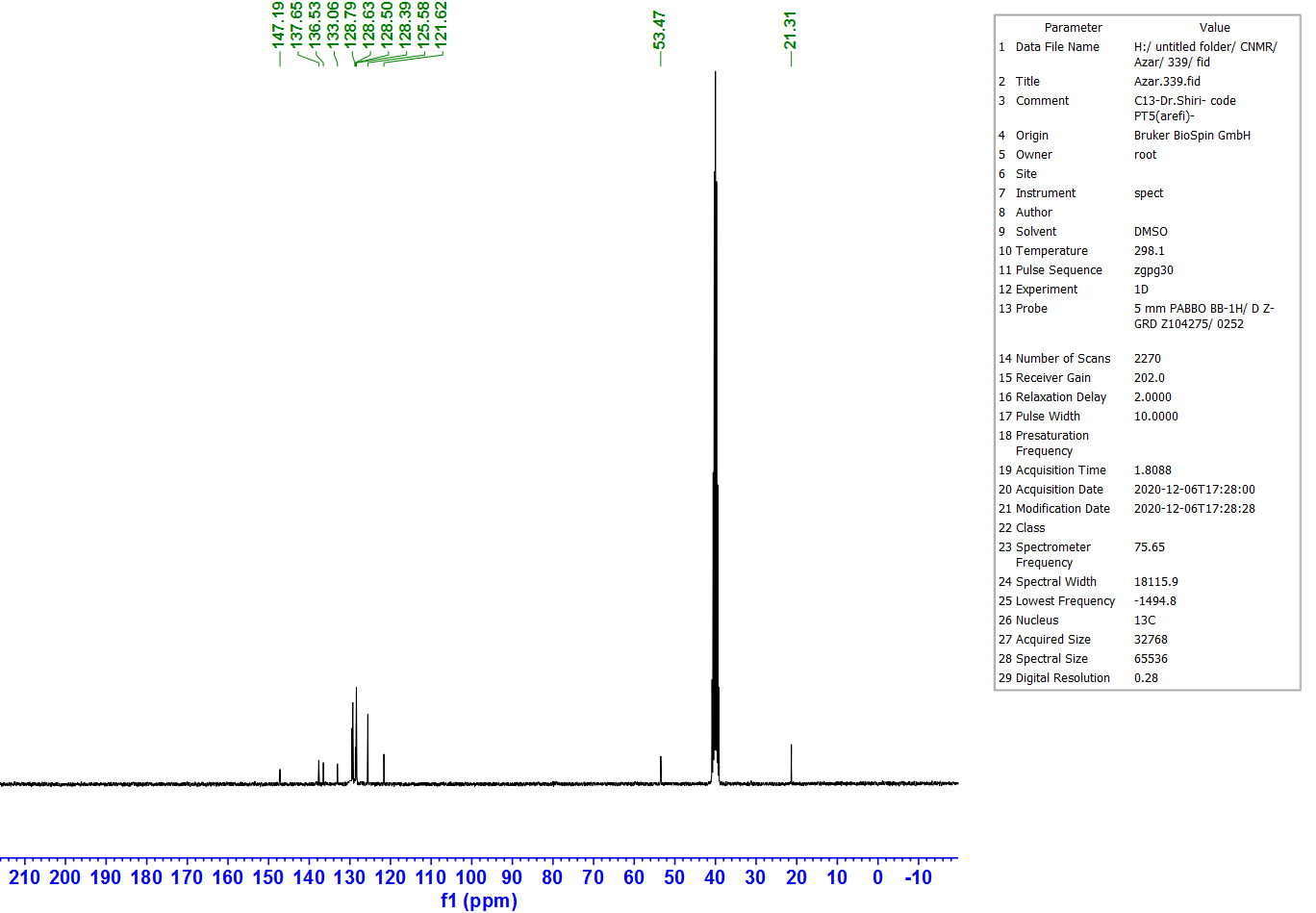


Fig. S13. ^13^C-NMR of 1-benzyl-4-p-tolyl-1,2,3-triazole (Table 2, compound 4h)

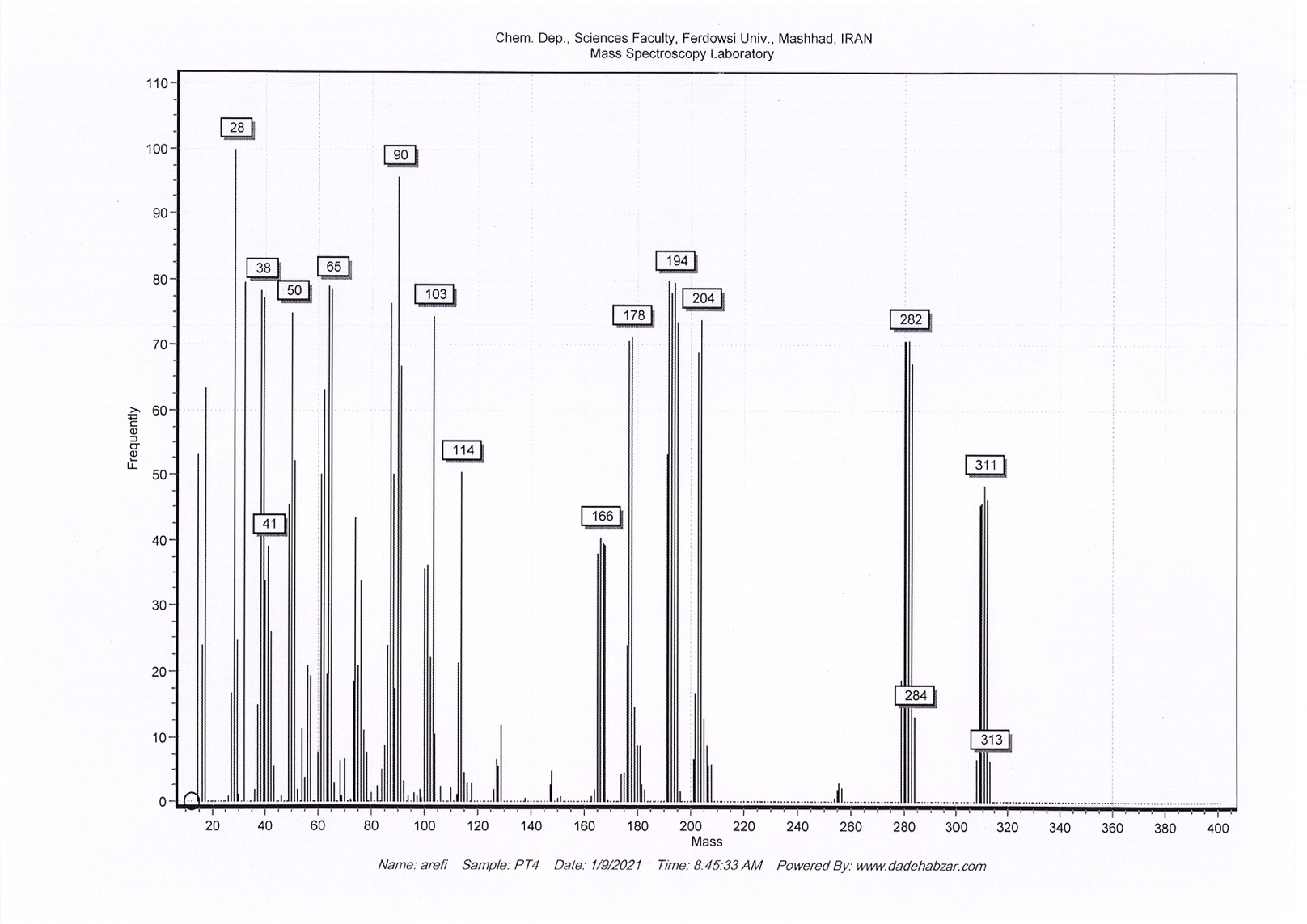


Fig. S14. Mass Spectrum of 1-benzyl-4-(4-bromophenyl)-1H-1,2,3-triazole (Table 2, compound 4i)

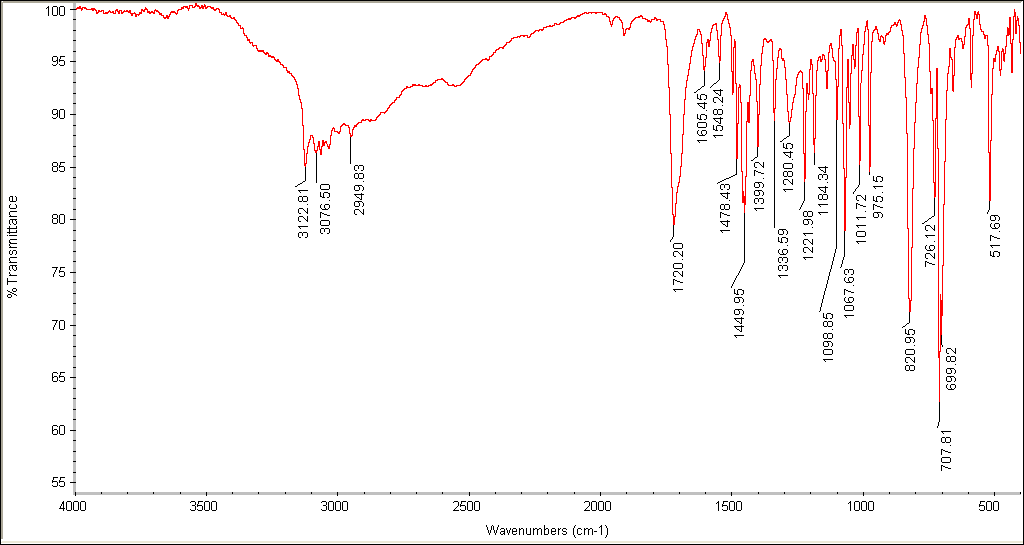


Fig. S15. FT-IR of 1-benzyl-4-(4-bromophenyl)-1H-1,2,3-triazole (Table 2, compound 4i)

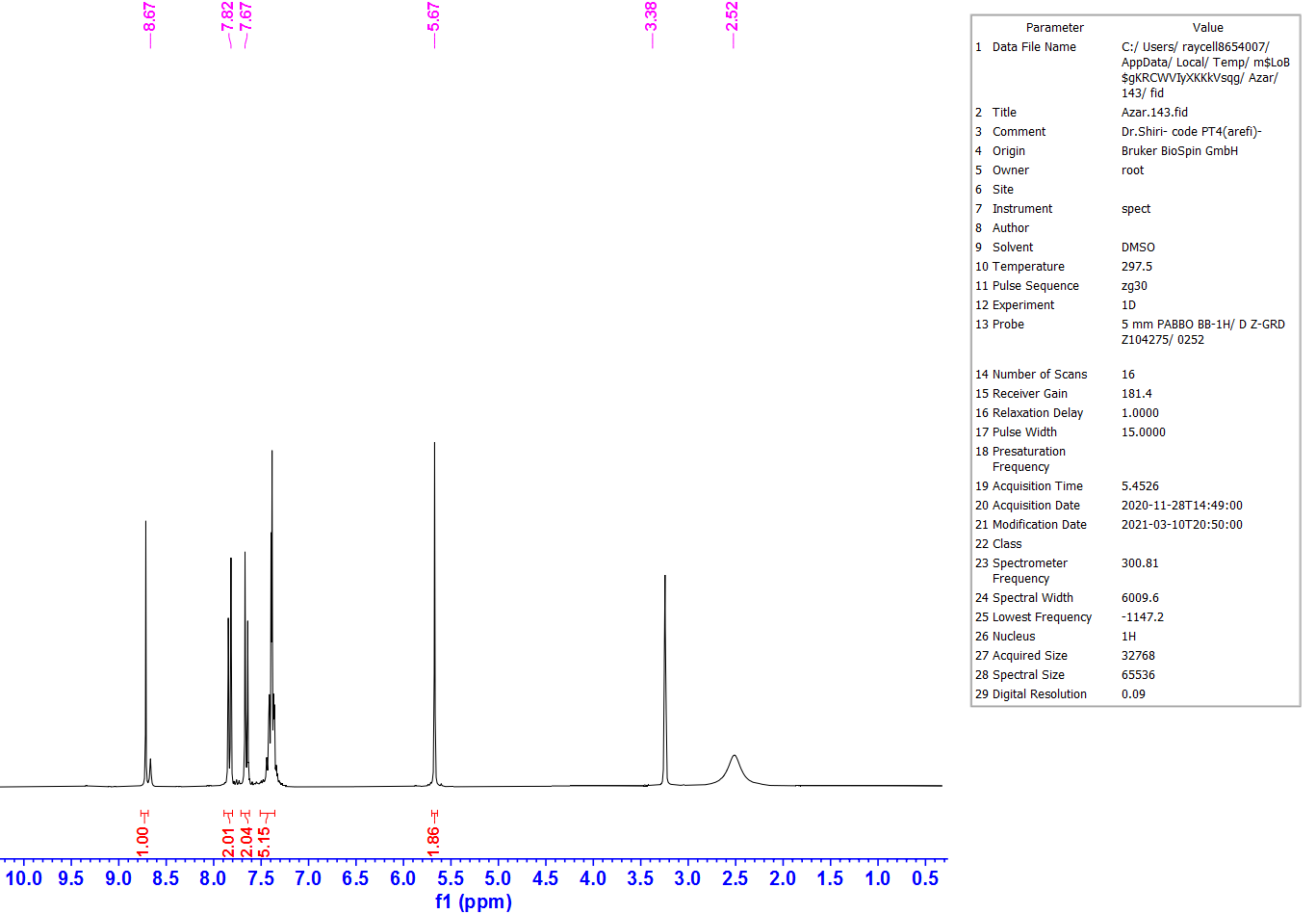


Fig. S16. ^1^H-NMR of 1-benzyl-4-(4-bromophenyl)-1H-1,2,3-triazole (Table 2, compound 4i)

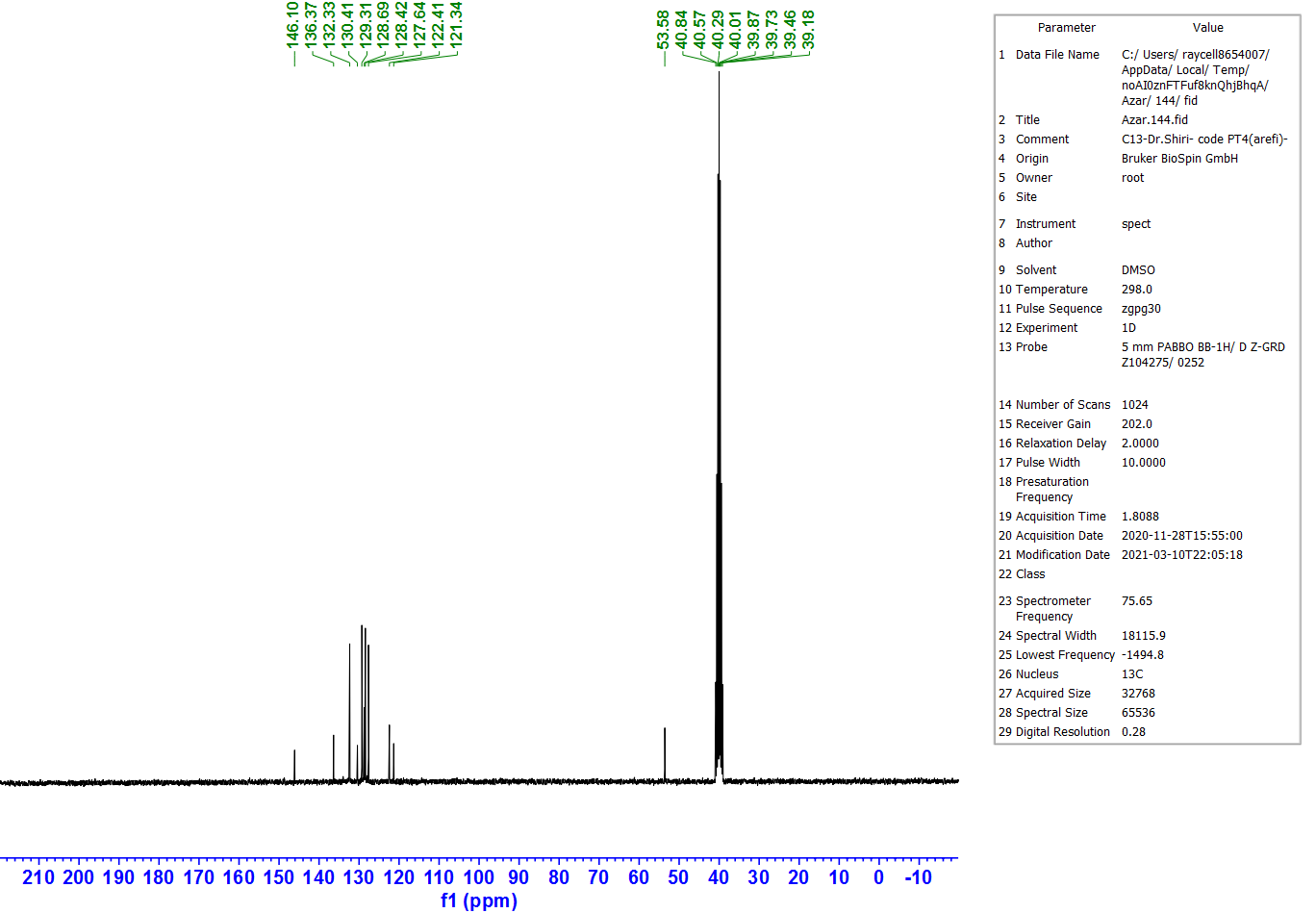


Fig. S17. ^13^C-NMR of 1-benzyl-4-(4-bromophenyl)-1H-1,2,3-triazole (Table 2, compound 4i)

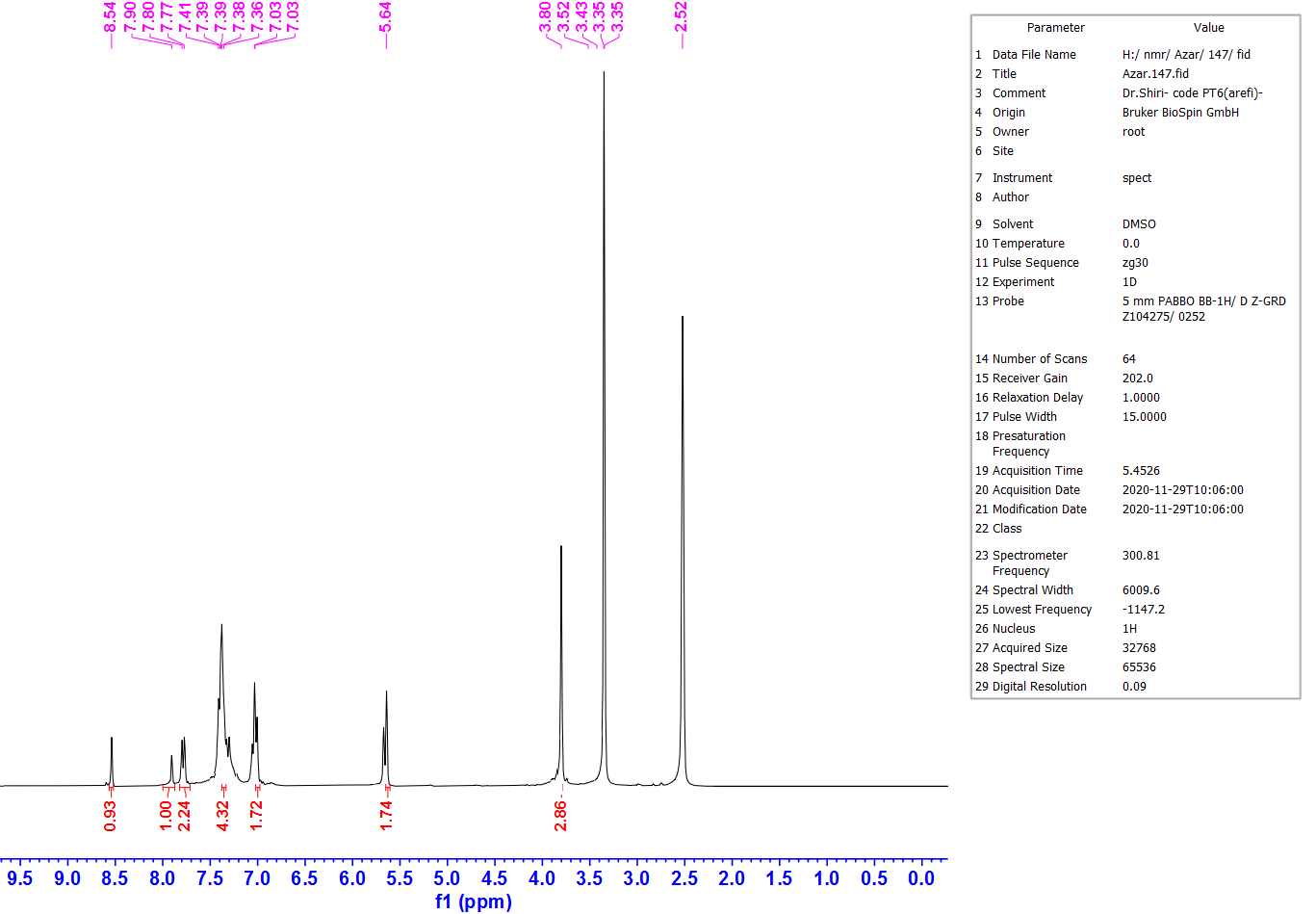


Fig. S18. ^1^H-NMR of 1-benzyl-4-(4-methoxyphenyl)-1H-1,2,3-triazole (Table 2, compound 4j)

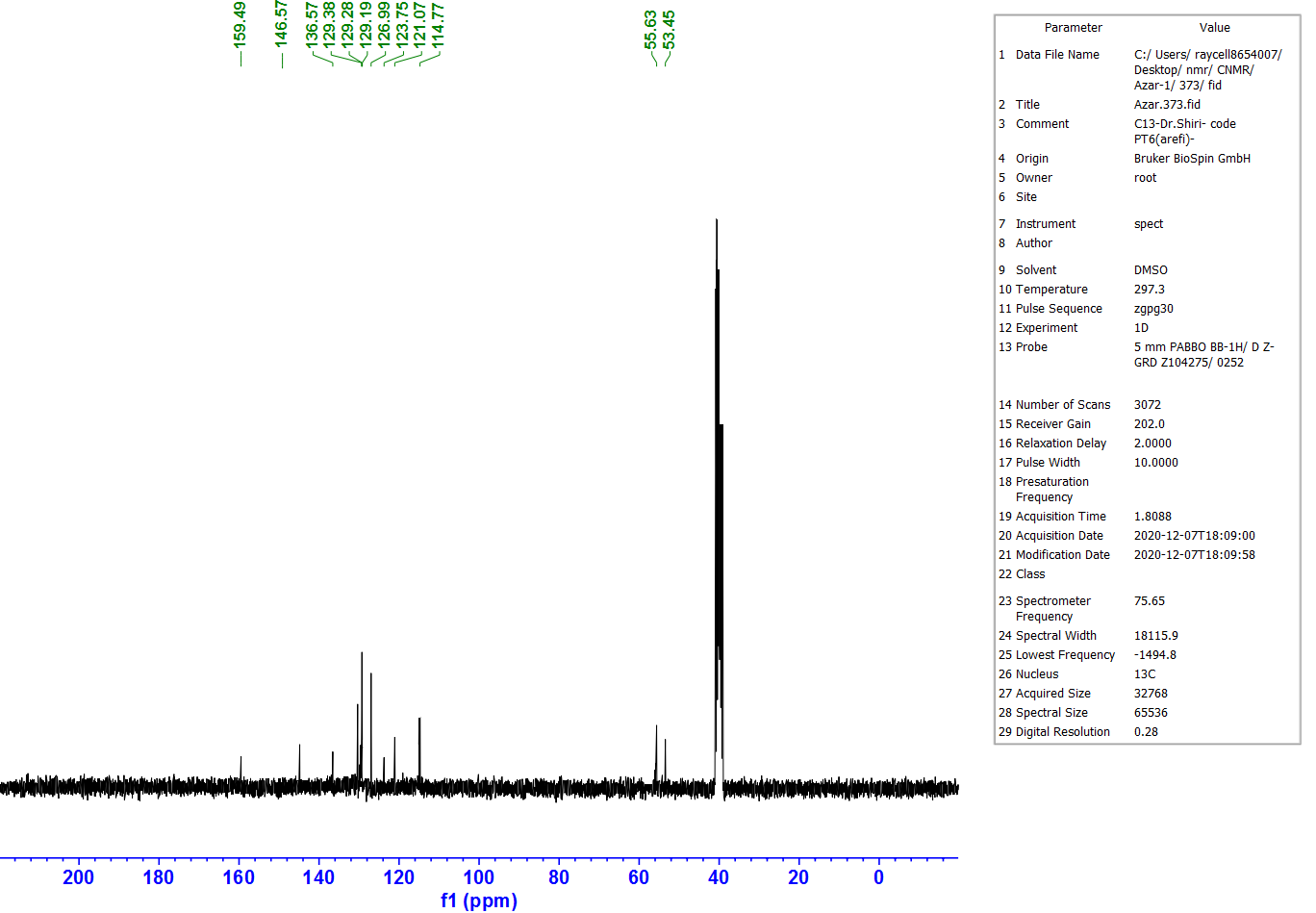


Fig. S19. ^13^C-NMR of 1-benzyl-4-(4-methoxyphenyl)-1H-1,2,3-triazole (Table 2, compound 4j)
